# High efficiency preparation of monodisperse plasma membrane derived extracellular vesicles for therapeutic applications

**DOI:** 10.1038/s42003-023-04859-2

**Published:** 2023-05-03

**Authors:** Claudio L. Alter, Pascal Detampel, Roman B. Schefer, Claudia Lotter, Patrick Hauswirth, Ramya D. Puligilla, Vera J. Weibel, Susanne H. Schenk, Wolf Heusermann, Melanie Schürz, Nicole Meisner-Kober, Cornelia Palivan, Tomaž Einfalt, Jörg Huwyler

**Affiliations:** 1 Department of Pharmaceutical Technology, University of Basel, Klingelbergstrasse 50, 4056 Basel, Switzerland; 2 Swiss Nanoscience Institute, University of Basel, Klingelbergstrasse 82, 4056 Basel, Switzerland; 3 Department of Chemistry, University of Basel, Mattenstrasse 24a, BPR 1096, 4058 Basel, Switzerland; 4 Imaging Core Facility, University of Basel, Spitalstrasse 41, 4056 Basel, Switzerland; 5 Department of Biosciences & Medical Biology, University of Salzburg, Hellbrunnerstrasse 34, 5020 Salzburg, Austria

**Keywords:** Drug delivery, Proteomics, Nanoparticles

## Abstract

Extracellular vesicles (EVs) are highly interesting for the design of next-generation therapeutics. However, their preparation methods face challenges in standardization, yield, and reproducibility. Here, we describe a highly efficient and reproducible EV preparation method for monodisperse nano plasma membrane vesicles (nPMVs), which yields 10 to 100 times more particles per cell and hour than conventional EV preparation methods. nPMVs are produced by homogenizing giant plasma membrane vesicles following cell membrane blebbing and apoptotic body secretion induced by chemical stressors. nPMVs showed no significant differences compared to native EVs from the same cell line in cryo-TEM analysis, in vitro cellular interactions, and in vivo biodistribution studies in zebrafish larvae. Proteomics and lipidomics, on the other hand, suggested substantial differences consistent with the divergent origin of these two EV types and indicated that nPMVs primarily derive from apoptotic extracellular vesicles. nPMVs may provide an attractive source for developing EV-based pharmaceutical therapeutics.

## Abstract (web summary)

Monodisperse nano plasma membrane vesicles (nPMVs) as extracellular vesicles (EVs) are prepared with high yield and their properties are compared in detail to native EVs, as an alternative source for EV-based therapy research.

## Introduction

**Fig. 1:**
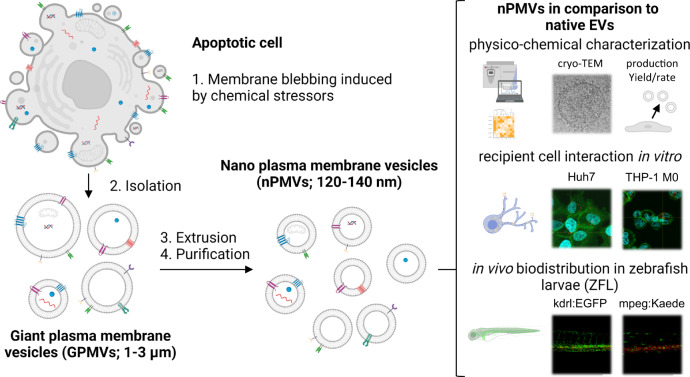
Schematic representation of the generation of nano plasma membrane vesicles (nPMVs), their characterization, and in vitro/in vivo studies for comparison to native extracellular vesicles (EVs). Donor cells are treated with chemical stressors (i.e., paraformaldehyde (PFA), dithiothreitol (DTT)) to induce apoptosis, which results in membrane blebbing and the production of ca. 1–3 µm sized apoptotic bodies (ApoBDs), namely giant plasma membrane vesicles (GPMVs). GPMVs are isolated and processed into unilamellar nPMVs through extrusion and purification to obtain a defined, monodisperse, and reproducible size distribution. nPMVs possibly differ in cargo and protein composition. The production rate and yield of the nPMV and native EV preparation method were evaluated. nPMVs were thereafter directly compared to native EVs using physico-chemical methods, 2D- and 3D-cryo-TEM, proteomics, lipidomics, in vitro recipient cell interactions using several human cell lines (i.e., Huh7 cells and THP-1 M0 macrophages), and biodistribution in transgenic (Tg) zebrafish larvae (ZFL) (i.e., Tg(kdrl:EGFP), Tg(mpeg:Kaede) (short for Tg(mpeg1:Gal4:UAS:Kaede)) as an in vivo vertebrate model.

Extracellular vesicles (EVs) are cell-derived membranous vesicles, which carry cell-specific lipids, proteins (i.e., enzymes, cell adhesion molecules, cluster of differentiation proteins (CD)), nucleic acids (i.e., various RNAs and DNAs), metabolites, organelles, and sometimes even viruses^1,2^. EVs are classified into two main categories based on their biogenesis: *exosomes and ectosomes*^2^. Exosomes are small EVs (40–150 nm diameter), originate from the endosomal compartment of the cell, and are continuously released to the surrounding by prokaryotic and eukaryotic cells through exocytosis of multivesicular bodies or amphisomes^2–4^. Ectosomes, on the other hand, form by direct budding/blebbing of the plasma membrane and comprise a large group of different types of vesicles including microvesicles, apoptotic bodies (ApoBDs), apoptotic extracellular vesicles (ApoEVs), and large oncosomes. Ectosomes have highly variable sizes ranging from 50 to >1000 nm^2,5,6^. Based on findings from proteomic and lipidomic analysis, EVs were shown to have a defined molecular fingerprint inherited from their donor (progenitor) cells^7,8^. In living organisms, they can interact with cells distant from their origin, be internalized to release their content, and induce phenotypic changes including alterations in signaling events^2,9,10^. For drug delivery purposes in cell and animal models, different strategies have been proposed for loading EVs with therapeutic nucleic acids, proteins, or small molecular weight drugs^11^. While most EV research in the field of therapeutic applications is still in a pre-clinical phase, a small set is progressing into human clinical trials^12,13^. Despite the great interest in EV (and in particular exosome) based technologies, progress slowed down not only, but in part due to challenges with production at scale. Typical EV secretion rates are as little as 60–170 particles/cell/h and production is highly dependent on the cell type used^14,15^. Thus, manufacturing large quantities of EVs requires substantial amounts of donor cells and is time-consuming. Additionally, isolation, purification, and sub-fractioning of the produced EVs are hampering the process^16^. The situation is further complicated by differences between protocols and experimental conditions used by individual laboratories. Therefore, guidelines for the standardization of production and characterization methods are being developed by the EV societies to accelerate pre-clinical research and ultimately the clinical translation of EV-based therapeutics^12,17^. Several approaches to increase the yield of EVs have been investigated. Mechanical extrusion or shear stress of whole cells generated higher yields of EV-like nanovesicles with physico-chemical and biological properties similar to native EVs^18,19^. Further, stressing of donor cells substantially increased the EV secretion in various models^20–24^. Due to limitations of standard laboratory EV preparation protocols, it was therefore the aim of the present study to develop an efficient method for the preparation of monodisperse EVs with high reproducibility. Giant plasma membrane vesicles (GPMVs) were produced by chemical stressors-induced cell apoptosis and subsequent membrane blebbing. GPMVs were explored to be used as a starting material (Fig. 1) for the production of EVs. Afterwards, the size of GPMVs was reduced by extrusion yielding nano plasma membrane vesicle (nPMV). nPMVs were then characterized side by side with native EVs isolated from the same donor cell line to compare their production yields and rates, physico-chemical properties, 2D- and 3D-morphology (using cryogenic transmission electron microscopy, cryo-TEM), molecular composition (proteomics and lipidomics), in vitro cellular interactions as well as in vivo biodistribution using transgenic (Tg) zebrafish larvae (ZFL) as a vertebrate pharmacokinetic model.

## Results

### Preparation of nPMVs

In the present study, we produced GPMVs as a starting material for the preparation of nPMVs and afterwards compared nPMVs to native EVs based on physico-chemical properties, cryo-TEM imaging, proteomics, lipidomics, and in vitro/in vivo studies (Fig. 1).

To maximize the GPMV yield, we first tested different chemical stressors (i.e., paraformaldehyde (PFA) and dithiothreitol (DTT), diallyl disulfide, n-ethylmaleimide) or an osmotic buffer for induction of membrane blebbing and GPMV production in HEK293 cells (Supplementary Fig. 1). PFA is a known cross-linking agent and induces cell shrinkage and separation of the plasma membrane form the cytoskeleton^25^. DTT is a disulfide-reducing agent. Together, these chemical stressors induce cortex contraction, subsequently, increase hydrostatic pressure, and ultimately lead to membrane blebbing^22^. We obtained the highest production rate of GPMVs (4–6 GPMVs/cell/h) with a combination of 25 mM PFA and 2 mM DTT for 6 h resulting in 24–36 GPMVs/cell with a diameter of 1–3 µm. Then, GPMVs were separated from residual cells and cell debris by centrifugation with 100 × *g*. GPMVs primarily comprise ApoBDs as observed by annexin V Alexa Fluor 488 binding (Supplementary Fig. 2). To convert GPMVs into nPMVs, we tested several size homogenization methods (i.e., microfluidic mixing, ultrasonication, and extrusion through polycarbonate filters) to reduce the hydrodynamic diameter (*D*_H_) of GPMVs from micrometer to nanometer scale (Supplementary Fig. 3). Microfluidic mixing and ultrasonication led to polydisperse preparations, whereas monodisperse nPMV preparations (polydispersity index (PDI) ≤ 0.2) were produced by extrusion of GPMVs through filter membranes with pore sizes of 50, 100, 200, or 400 nm. The size of nPMVs was highly dependent on the pore size of the extrusion filter membranes. Furthermore, we adapted the extrusion process to a pressurized barrel extruder for higher process volumes (1–100 mL) and scaled up production. We observed no differences in *D*_H_ and PDI between the two approaches (Supplementary Fig. 4). All subsequent studies were performed with nPMVs extruded through 100 nm extrusion filter membranes and purified by dialysis to remove residual PFA and DTT.

### Physico-chemical characterization and storage stability

**Fig. 2:**
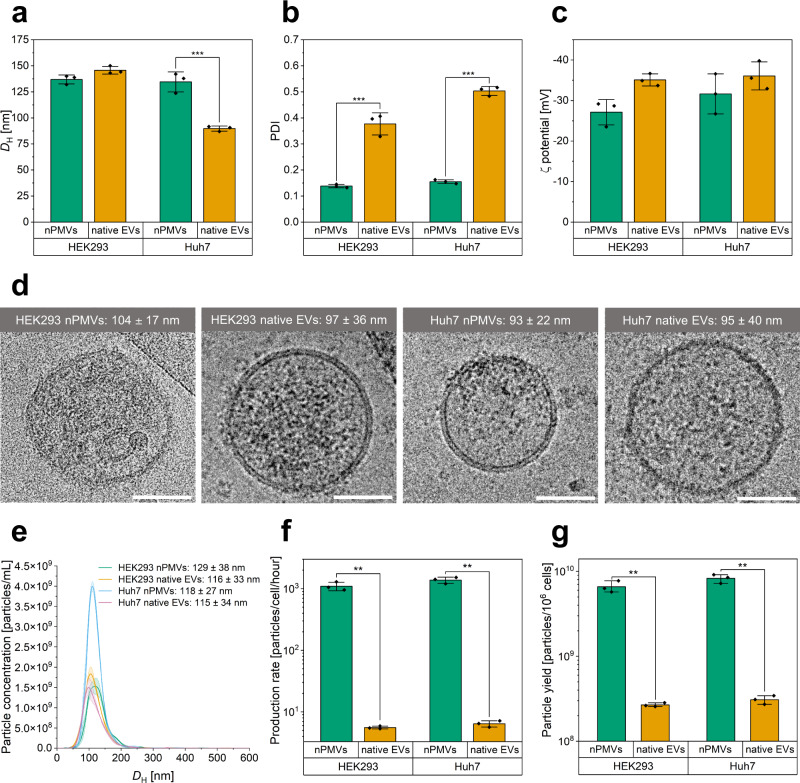
Physico-chemical characterization, cryo-TEM analysis, and nanoparticle tracking analysis (NTA) of HEK293 and Huh7 nPMVs and native EVs. Comparison of production rates and yields of the nPMV and native EV preparation protocols. Hydrodynamic diameter (D_H_) (**a**), polydispersity index (PDI) (**b**), and ζ potential (**c**) of HEK293 and Huh7 nPMVs and native EVs. Values are means ± SD, squares: data points, *n* = 3 measurements. **d** Representative cryo-TEM images of HEK293 and Huh7 nPMVs and native EVs including their diameter. Scale bar: 50 nm. Values are means ± SD, *n* ≥ 15 vesicles. **e** Nanoparticle (NP) concentration as function of the D_H_ size distribution of HEK293 nPMVs (green), HEK293 native EVs (orange), Huh7 nPMVs (blue), and Huh7 native EVs (pink) measured by NTA. Values are means ± SD, *n* = 3 measurements. **f** Production rate (particles/cell/hour) of HEK293 and Huh7 nPMVs and native EVs produced by the nPMV or the native EV preparation protocol. **g** Theoretical total particle yield of the nPMV or native EV preparation protocol obtained from 10^6^ donor cells in 6 or 48 h, respectively. This results in a 27-fold improvement in particle yield and fourfold reduction in production time. Values are means ± SD, squares: data points, *n* = 3 measurements. Levels of significance: *p ≤ 0.05, **p ≤ 0.01, ***p ≤ 0.001.

For the comparison study between nPMVs and native EVs, we decided to use HEK293 and Huh7 as commonly used donor cells for EVs. Purification was by size exclusion chromatography as recommended previously^12,26,27^. We evaluated colloidal stability of these nPMVs and native EVs by measuring their *D*_H_, PDI, and ζ potential (*n* = 3 measurements). We observed comparable *D*_H_ for HEK293 nPMVs (137 ± 4 nm) and native EVs (146 ± 4 nm) (Fig. 2a). According to dynamic light scattering, Huh7 nPMVs had a *D*_H_ of 135 ± 10 nm while Huh7 native EVs were smaller (*D*_H_ of 90 ± 2 nm). HEK293 and Huh7 nPMVs were monodisperse (PDI < 0.16) but native EVs polydisperse (PDI = 0.37–0.5) preparations (Fig. 2b). ζ potentials of all preparations were comparable (−29 to −36 mV) (Fig. 2c). In addition, we prepared A549, HepG2, and THP-1 M0 nPMVs to demonstrate the applicability of our optimized preparation protocol to other cell lines and measured no significant difference in *D*_H_, PDI, or ζ potentials compared to HEK293 and Huh7 nPMVs (Supplementary Fig. 5). HEK293 and Huh7 nPMVs and native EVs were imaged using cryo-TEM to evaluate their structure, shape, and the presence of macromolecules (Fig. 2d). All preparations had a predominantly globular shape formed by a phospholipid bilayer (4.5 ± 0.9 nm thickness) and negligible fractions of multilamellar vesicles were observed. We measured vesicle diameters (*n* ≥ 15 vesicles) of 104 ± 17 nm for HEK293 nPMVs, 97 ± 36 nm for HEK293 native EVs, 93 ± 22 nm for Huh7 nPMVs, and 95 ± 40 nm for Huh7 native EVs, respectively. All preparations showed vesicles with different amounts of macromolecules on the surface/membrane and inside the core. Cryo-TEM images of A549, HepG2, and THP-1 M0 nPMVs are represented in Supplementary Fig. 6a. To further visualize the presence of membrane proteins, we performed a 3D image reconstruction (tomography) of a THP-1 M0 nPMV (Supplementary Fig. 6b). To study stability over time, freshly prepared HEK293 and Huh7 nPMVs and native EVs were stored at 4 °C or room temperature for 1 month (Supplementary Fig. 7). Samples stored at 4 °C were stable up to 1 month after production as assessed based on *D*_H_ and PDI and the colloidal stability of nPMVs and native EVs was comparable. In contrast, nPMV samples stored at room temperature had an increased *D*_H_ and PDI after 1 month of storage.

Nanoparticle tracking analysis (NTA) measurements were performed to investigate the particle concentration as a function of the *D*_H_ size distribution (Fig. 2e). *D*_H_ averaged at 129 ± 38 nm for HEK293 nPMVs, 116 ± 33 nm for HEK293 native EVs, 118 ± 27 nm for Huh7 nPMVs, and 115 ± 34 nm for Huh7 native EVs, respectively. We calculated the production yields and rates for the nPMV and native EV preparation protocols using the particle concentrations, sample volumes, and cell numbers at the starting point of the particle production (Supplementary Table 1).

We calculated a production rate of 1100–1386 particles/cell/hour for the nPMV and 5–6 particles/cell/hour for the native EV preparation protocol (*p* ≤ 0.01), respectively. Additionally, we observed a significantly higher production yield for our nPMV preparation protocol (6600–8320 particles/cell) compared to the native EV preparation protocol (270–310 particles/cell) (*p* ≤ 0.01). Of note, nPMVs were recovered after 6 h whereas for native EVs medium was conditioned for 48 h. Using the nPMV preparation protocol, a 27-fold increase in total particle yield and a fourfold reduction in production time is achieved compared to the native EV preparation protocol, with up to 8.3 × 10^9^ or 3.1 × 10^8^ particles obtained from 10^6^ donor cells (Fig. 2f, g). Given 48 h of total production time and access to additional cells, the use of the nPMV preparation protocol could result in a total yield of up to 3.3 × 10^10^ nPMVs, which would be 108 times higher than the yield obtained with the EV preparation protocol (3.1 × 10^8^ particles).

We further analyzed the ratio of particles per ug protein using the NTA particle and average protein concentration. 4.0 ± 0.4 × 10^11^, 13.5 ± 0.4 × 10^11^, 8.1 ± 0.6 × 10^11^, and 4.6 ± 0.3 × 10^11^ particles per ug protein were calculated for HEK293 nPMVs, HEK293 native EVs, Huh7 nPMVs, and Huh7 native EVs, respectively. The inverted protein/particle amounts were: 2.50 ± 0.02 pg/particle for HEK293 nPMVs, 0.74 ± 0.17 pg/particle for HEK293 native EVs, 1.25 ± 0.02 pg/particle for Huh7 nPMVs, and 2.18 ± 0.04 pg/particle for Huh7 native EVs.

### Proteomic and lipidomic characterization of nPMVs

**Fig. 3:**
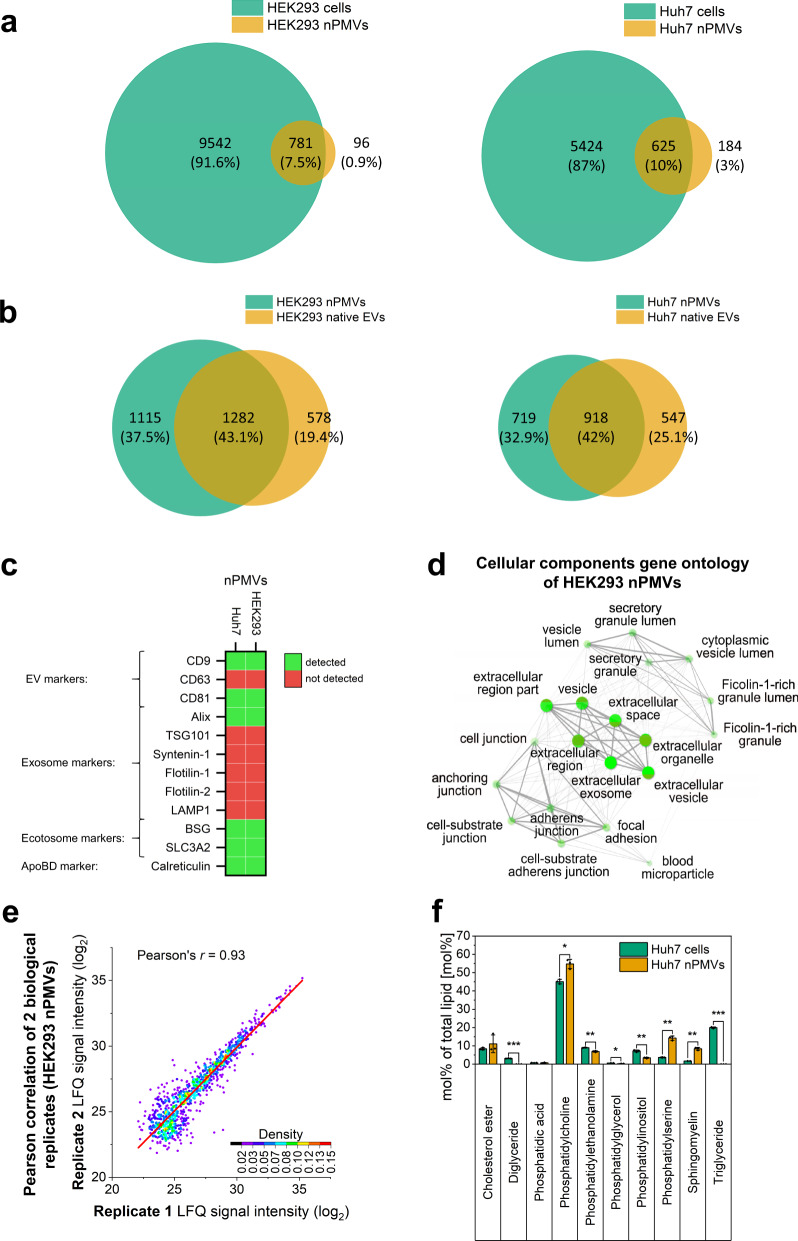
Proteomic analysis of nPMVs, donor cells, and native EVs, reproducibility of nPMV proteome, and lipidomics of Huh7 nPMVs and donor cells. **a** Venn diagram for comparison of HEK293 (left) or Huh7 (right) nPMVs proteome (orange) and the corresponding donor cell line proteome (green). Size of the circle is representative for the total counts. Overlapped circle area is representative for the overlap counts. **b** Venn diagram for comparison of the proteome of HEK293 or Huh7 nPMVs (green) with the corresponding native EVs (orange). Size of the circle is representative for the total counts. Overlapping circle area is representative for the overlap counts. **c** Heatmap illustrating the presence of EV, exosome, ectosome, and ApoBD markers in HEK293 and Huh7 nPMVs. Green: detected in the proteome. Red: not detected in the proteome. **d** Representative cellular component gene ontology analysis of the top 50 proteins of HEK293 nPMVs using the ShinyGO database. Bright green spots indicate enrichment. **e** Pearson correlation between the label-free quantification (LFQ) of biological replicate 1 and 2 of HEK293 nPMVs. Pearson correlation of Huh7 is represented in Supplementary Fig. 14. The whole proteome was used for the analysis. Color map: indicates the dot plot density. Red line: linear fit of the data. Detailed findings of the proteomic analysis are provided as Supplementary Figs. 8–15 and Supplementary Tables 2 and 3. **f** Lipidomic analysis of Huh7 nPMVs (orange) and Huh7 donor cells (green). Values are means ± SD, squares: data points, *n* = 3. Levels of significance: *p ≤ 0.05, **p ≤ 0.01, ***p ≤ 0.001.

First, the proteomes of HEK293 or Huh7 nPMVs were compared to their donor cells. Around 7% and 10% of the corresponding donor cell proteins were identified in the proteome of HEK293 and Huh7 nPMVs (Fig. 3a). On the other hand, between 80 and 90% of the nPMV proteins were overlapping with the corresponding donor cell proteome.

When directly comparing proteomes of either HEK293 or Huh7 nPMV and native EVs, we observed an overlap of 43.1% for HEK293 and 42% for Huh7 between the corresponding nPMVs and native EVs, including various damage-associated molecular patterns (DAMPs) proteins (Fig. 3b). Unique proteins found in nPMVs included several caspases, which are key factors of apoptosis. In contrast, unique proteins for native EVs were found to be primarily associated with lysosomal and endocytosis pathways. Interestingly, based on the proteomics data, we also measured around 30% and 12% higher amounts of total individual protein identities in HEK293 and Huh7 nPMVs compared to corresponding native EVs for HEK293 and Huh7 cell, respectively. From three main tetraspanins typically enriched in EVs (i.e., CD9, CD63, CD81), we identified CD9 and CD81 in HEK293 and Huh7 nPMVs (Fig. 3c). However, CD63 was not detected in any of the nPMV preparations. From the commonly used exosome markers (i.e., Alix, TSG101, Syntenin-1)^14^, which are proteins associated with the endosomal sorting complex required for transport (ESCRT) and important for exosome biogenesis, only Alix was detected HEK293 and Huh7 nPMVs. Exosome markers flotillin-1/2 and LAMP1 were not found, whereas both recently published ectosome (i.e., BSG, SLC3A2)^28^ and ApoBD (i.e., calreticulin)^29^ markers were detected in the nPMV proteomes.

We further compared the proteomes of five different nPMV types (A549, HEK293, HepG2, Huh7, and THP-1 M0) and identified 466 common proteins, composing likely a set of common nPMV proteins (Supplementary Fig. 8). These included known EV proteins such as chaperones, 14-3-3 proteins, cytoskeletal proteins, annexins, enzymes, and several CD proteins (i.e., CD71, CD98, CD147 (BSG))^7,8,30,31^. The complete list of identified EV proteins is provided in Supplementary Table 1. We also observed various DAMPs (i.e., S100 proteins, heat shock proteins, F-actin components, histones, and peroxiredoxins)^32^. A detailed list of DAMP proteins, which are also identified in EVs, can be found in Supplementary Table 2. The common nPMV marker proteins were then compared against the top 100 reported EV markers of Vesiclepedia^30,33^ and ExoCarta^31,34^ databases, which are compendiums of molecular EV data (i.e., lipids, proteins, and RNA). Within the 466 common nPMV marker proteins, we identified 62 proteins in the top 100 EV markers of Vesiclepedia and 55 of ExoCarta, whereas 395 proteins were unique for nPMVs (Supplementary Fig. 9).

In addition to the common nPMV proteins, distinct differences between the proteomes of the five different nPMV types were detected: First, when directly comparing HEK293 or Huh7 nPMV proteins to the 466 common nPMV and the corresponding donor cell proteins, an overlap between nPMVs and the corresponding donor cell line was observed (Supplementary Fig. 10). Second, differentially expressed proteins were detected between the different nPMV types when heat mapping 150 proteins of the common 466 nPMV proteins (Supplementary Fig. 11) and comparing HepG2 and Huh7 nPMV proteomes (Supplementary Fig. 12a). This difference in expression was even more evident when proteomes between Huh7 and THP-1 M0 nPMVs were compared (Supplementary Fig. 12b).

For each of the five nPMV types, the top 50 abundant proteins based on intensity-based absolute quantification (iBAQ) were analyzed for cellular components gene ontology (GO) with ShinyGO^35^. The iBAQ value is the sum of all identified peptide intensities divided by the number of theoretically detectable peptides^36^. The top 50 abundant proteins of one nPMV type make up >50% of the total protein mass detected in its proteome. GO networks were similar for all nPMV types and showed a strong enrichment in several extracellular components (Fig. 3d shows the representative GO network of HEK293 nPMVs): First, enrichment (*n* = 2324, *p* value = 1.2 × 10^−36^) in extracellular vesicle proteins^37^, which include proteins of any vesicle that is part of the extracellular region. Second, in extracellular organelle proteins^38^ (*n* = 2326, *p* value = 1.2 × 10^−36^), which are proteins found in an organized structure of distinctive morphology and function outside of the cell (i.e., extracellular vesicles). Third, in extracellular exosome proteins^39^ (*n* = 2300, *p* value = 1.2 × 10^−36^), which are proteins found in exosomes. Moreover, we discovered enrichment in cell adhesion proteins, secretory, and ficolin-1-rich components. Thereby, these data further confirmed the enrichment of EV-associated proteins in nPMVs.

Next, we were interested in the proteome reproducibility of HEK293 and Huh7 nPMVs. When comparing the proteomes of three replicates, we found an overlap of 84.6% for HEK293 and 83.6% for Huh7 nPMVs (Supplementary Fig. 13). Additionally, we observed Pearson’s *r* values of 0.9 or 0.93 when comparing the label-free quantification (LFQ) of the whole proteomes of replicate 1 with replicate 2 of HEK293 or Huh7 nPMVs (Fig. 3e and Supplementary Fig. 14), respectively. Of interest, we analyzed a fourth replicate half a year later in order to stress test the practical reproducibility of the nPMV preparation method. Pearson’s *r* values of 0.92 and 0.83 were calculated for HEK293 and Huh7, respectively, thereby underlining the high reproducibility of the preparation method (Supplementary Fig. 15).

Presentation of lipids (e.g., phosphatidylserine (PS)) is widely exploited by enveloped viruses (i.e., EBOLA, Dengue) and parasites as an essential co-factor for endocytic cell entry^40–44^, and has also been proposed to be used by exosomes for cell entry^26^. For this reason, we were interested in analyzing the lipidome of Huh7 cells and Huh7 nPMVs by shotgun MS-lipidomics^45^ (Fig. 3f). PS, sphingomyelin (SM), and phosphatidylcholine were statistically significantly (*p* ≤ 0.01) enriched in nPMVs as compared to the donor cells. The PS content in nPMVs was increased (14.3 mol% of total lipid) as compared to the donor cells (3.7 mol%). The lipid content of phosphatidylethanolamine, phosphatidylglycerol, and phosphatidylinositol was lower in nPMVs compared to donor cells (*p* ≤ 0.05). The di- and triglycerides were present in host cells, but absent in nPMVs (*p* ≤ 0.001).

Based on these lipidomic findings, we decided to produce two synthetic liposomal formulations: pure 1,2-dioleoyl-sn-glycero-3-phosphocholine (DOPC) and DOPC:PS liposomes with similar *D*_H_ to nPMVs and native EVs. These liposomal formulations will serve as PS-negative (DOPC) and PS-positive (DOPC:PS) synthetic controls in comparison to nPMVs and native EVs to assess the contribution of PS to cell uptake in in vitro and in vivo models. To confirm the presence of PS on the outer surface of HEK293 and Huh7 nPMVs and native EVs, and the liposomal formulations, we investigated binding of the PS-specific annexin V protein to these NPs by fluorescence correlation spectroscopy (FCS) using an annexin V Alexa Fluor 488 conjugate^46^ (*D* = 90 ± 0.01 μm^2^/s). We observed distinct diffusion time shifts when annexin V Alexa Fluor 488 conjugate was incubated with nPMVs and native EVs (*D* = 3.7–6.2 μm^2^/s) and DOPC:PS liposomes (*D* = 5 ± 0.02 μm^2^/s). No relevant shift (*D* = 95 ± 0.01 μm^2^/s) was detected for the negative control (DOPC liposomes, Supplementary Fig. 16).

### Tolerability assessment and in vitro cellular interaction studies

**Fig. 4:**
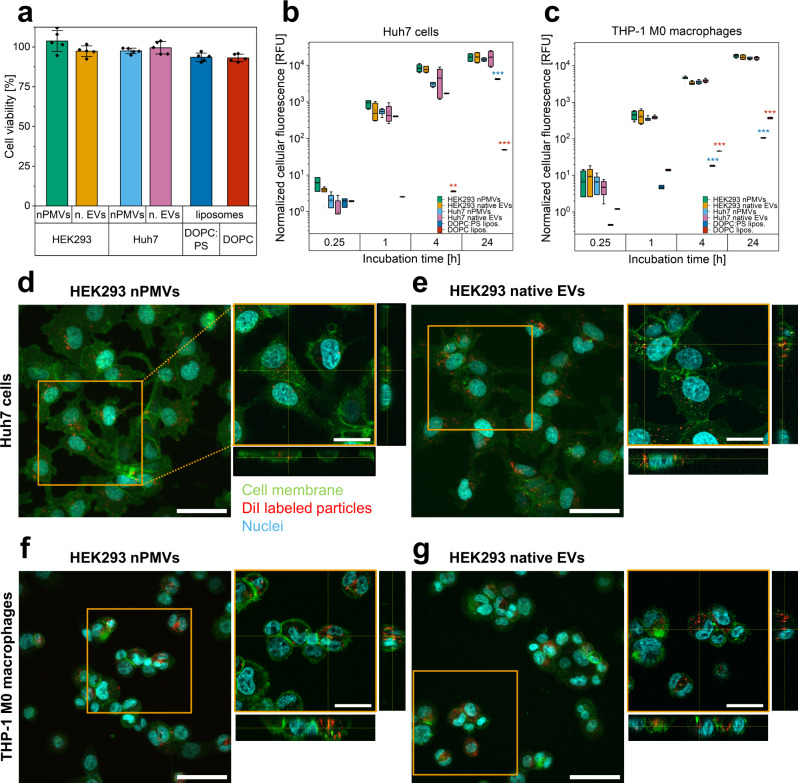
Viability assessment and in vitro cellular interactions of HEK293 and Huh7 nPMVs and native EVs, DOPC:PS, and DOPC liposomes with Huh7 cells and THP-1 M0 macrophages. **a** Huh7 cell viability with HEK293 and Huh7 nPMVs and native EVs, DOPC:PS, and DOPC liposomes measured by MTS assay after 24 h incubation. Values are means ± SD, squares: data points, *n* = 3. **b**, **c** Flow cytometry based cellular uptake quantification of 1,1 dioctadecyl-3,3,3′,3′-tetramethylindocarbocyanine (DiI) labeled HEK293 nPMVs (green), HEK293 native EVs (orange), Huh7 nPMVs (blue), Huh7 native EVs (pink), DOPC:PS (dark blue), and DOPC (red) liposomes by Huh7 cells (**b**) and THP-1 M0 macrophages (**c**) after 0.25, 1, 4, and 24 h incubation. Box plot: line: median, square: mean, box: lower and upper quartile, whisker: 1.5 interquartile range, filled square: outlier. Values are the normalized cellular fluorescence units (RFU), which were normalized using the NP brightness of each individual NP formulation as measured by FCS (counts per molecule, Supplementary Table 4), *n* = 3. **d**, **e** Z-projection of Huh7 cells with HEK293 nPMVs (**d**) and HEK293 native EVs (**e**) imaged with confocal laser scanning microscopy (CLSM) after incubation for 1 h. Cyan signal: nuclei. Green signal: cell membrane. Red signal: DiI labeled NPs. Scale bar: 50 µm. Right panel: Zoomed in Z-projection and orthogonal view of the orange indicated region. Scale bar: 25 µm. **f**, **g** Incubation of HEK293 nPMVs and native EVs with THP-1 M0 macrophages. Same experimental setup as in **d** and **e**. Corresponding CLSM images of Huh7 nPMVs and native EVs, DOPC:PS, and DOPC liposomes with Huh7 cells and THP-1 M0 macrophages including untreated control cells are shown in Supplementary Fig. 19. Levels of significance: *p ≤ 0.05, **p ≤ 0.01, ***p ≤ 0.001.

It was previously shown that EVs can promote or inhibit cell growth and proliferation^47–49^. In order to assess the tolerability of our nPMVs, native EVs, and liposomal formulations, the cell viability of Huh7 cells was determined in response to exposure to NPs using the 3-(4,5-dimethylthiazol-2-yl)-5-(3-carboxymethoxyphenyl)-2-(4-sulfophenyl)-2H-tetrazolium (MTS) assay. These experiments demonstrated no significant cell viability change after 24 h incubation with NPs as compared to untreated control cells (Fig. 4a). In contrast, statistically significantly (*p* < 1 × 10^−19^) lower cell viabilities of 35–45% were observed for cells exposed to non-dialyzed HEK293 and Huh7 nPMVs which still contained residual PFA and DTT (Supplementary Fig. 17).

To quantitatively and qualitatively monitor cellular interactions and uptake dynamics of HEK293 and Huh7 nPMVs and native EVs, DOPC:PS, and DOPC liposomes, a combination of flow cytometry and confocal laser scanning microscopy (CLSM) based live cell imaging was used. For both studies, we used 1,1-dioctadecyl-3,3,3′,3′-tetramethylindocarbocyanine (DiI) labeled NPs, nPMVs, and native EVs were labeled following the same protocol. We additionally found that all NPs had similar brightnesses (Supplementary Table 4). For the flow cytometry studies, DiI-labeled NPs were incubated with Huh7 cells and THP-1 M0 macrophages for 0.25, 1, 4, and 24 h. After incubation, the median relative fluorescence units (RFU) of these cells were analyzed using flow cytometry and normalized using the brightness of each individual NP formulation as measured by FCS (Fig. 4b, c). We observed a time-dependent increase in normalized cellular fluorescence for all NPs, which was highest during the first hour of incubation and reached saturation after 4 h. No significantly different normalized cellular fluorescence of Huh7 cells or THP-1 M0 macrophages incubated with HEK293 or Huh7 nPMVs or native EVs was seen at any time point. In Huh7 cells, the interaction of HEK293 and Huh7 nPMVs and native EVs was more than three-fold higher (p ≤ 0.001) compared to DOPC:PS liposomes after 24 h incubation, whereas in THP-1 M0 macrophages a 15–18-fold higher normalized cellular fluorescence was observed (*p* ≤ 0.001). Interaction of DOPC liposomes was up to 50–300-fold lower than nPMVs and native EVs on both tested cell lines (*p* ≤ 0.001). To confirm internalization and qualitatively monitor the subcellular localization of the different NPs, we imaged Huh7 cells and THP-1 M0 macrophages after incubation with NPs for 1 h using CLSM. Consistent with the previously elucidate canonical uptake routes for CD63 positive exosomes^26^, we observed single HEK293 or Huh7 nPMVs or native EVs or DOPC:PS liposomes on the cell surface and on filopodia of both cell lines after a few minutes of incubation. Within the next 15–30 minutes, the amount of these NPs increased drastically. After 1 h, the highest signals were observed for HEK293 and Huh7 nPMVs and native EVs followed by DOPC:PS liposomes in both tested cell lines (Fig. 4d–g, Supplementary Fig. 19). Negligible internalization of DOPC liposomes was observed. We detected internalization of all NPs but DOPC liposomes in both cell lines by 3D stacks and orthogonal views (right panel) of the orange indicated regions (Fig. 4d–g, Supplementary Fig. 19). CLSM results were in line with flow cytometry cell interaction results. To evaluate intracellular movement kinetics, all NPs were incubated with either Huh7 cells or THP-1 M0 macrophages and imaged over 30 min (Supplementary Fig. 20). In both recipient cell lines, HEK293 and Huh7 nPMVs and native EVs showed similar single particle trajectories, intracellular mean NP trafficking speeds (0.09–0.12 μm/s), and distribution patterns. Interestingly, DOPC:PS liposomes had a significantly lower mean NP trafficking speed (0.05 ± 0.02 μm/s, *p* ≤ 0.001). In contrast, for DOPC liposomes no interaction with Huh7 cells were observed after 30 min. Furthermore, we observed colocalization with lysosomes using Lysotracker Green as a marker for acidified compartments, for all NP except DOPC liposomes with Huh7 cells 4 h after incubation (Supplementary Fig. 21). Similar results were obtained for nPMVs and native EVs on THP-1 M0 macrophages, however DOPC:PS liposomes showed lower colocalization after 4 h incubation. To understand mechanisms of cellular uptake, DiI labeled HEK293 and Huh7 nPMVs and native EVs, DOPC:PS, and DOPC liposomes were incubated with Huh7 cells in presence or absence of known transport inhibitors at non-toxic concentrations, as tested with MTS assay, and analyzed by flow cytometry (Supplementary Figs. 22, 23). Overall, the uptake of nPMVs showed a similar susceptibility pattern to endocytosis inhibitors as the uptake of native EVs. Annexin (PS-binding protein), chlorpromazine (clathrin-mediated endocytosis inhibitor^50^), colchicine (micropinocytosis inhibitor^50^), polyinosinic acid (scavenger receptors inhibitor^51^), and sodium azide (NaN_3_) (mitochondrial respiration inhibitor, blocks energy-dependent endocytosis^52^) had a statistically significant influence on the uptake of both, HEK293 and Huh7 nPMVs and native EVs (*p* ≤ 0.05), whereas cytochalasin B (actin-dependent endocytosis inhibitor^53^), nystatin (caveolin-mediated endocytosis inhibitor^54^), and T-cell immunoglobulin and mucin-1 (TIM-1) antibody (blocks TIM-1 receptors) had no significant effect. Additionally, annexin V significantly lowered the uptake of DOPC:PS, but not DOPC liposomes. The uptake of DOPC:PS and DOPC liposomes was inhibited by NaN_3_ (*p* ≤ 0.001). TIM-1 antibody did not show any significant effects on the uptake of DOPC:PS or DOPC liposomes. Of note, flow cytometry experiments cannot distinguish between uptake and binding of NPs to cells^26^. Therefore, cells were extensively washed prior to flow cytometry analysis to remove surface-bound particles.

### In vivo biodistribution studies in ZFL

**Fig. 5:**
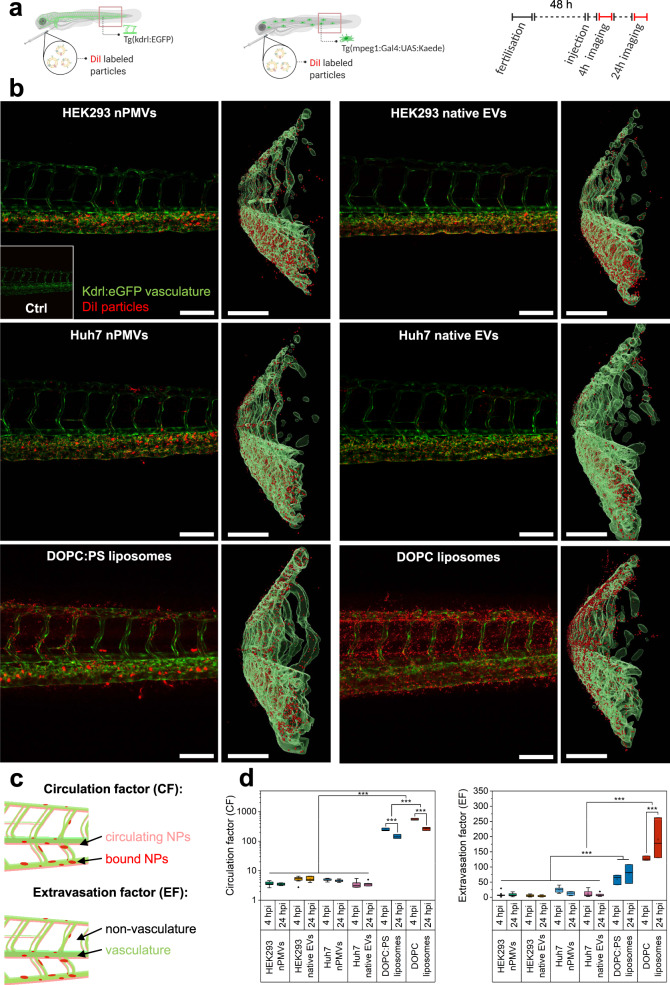
In vivo biodistribution of HEK293 and Huh7 nPMVs and native EVs, DOPC:PS, and DOPC liposomes in the zebrafish larvae (ZFL). **a** Schematic representation of the experimental procedure: DiI labeled NPs were injected into the duct of Cuvier of two transgenic ZFL fish lines (kdrl:EGFP and mpeg1:Gal4:UAS:Kaede) 48 h post fertilization. 4 and 24 h post injection (hpi), tissue distribution in the tail region (red rectangle) was visualized by CLSM. **b** Light panel: Tissue distribution of DiI labeled NPs (red signal) in Tg(kdrl:EGFP) ZFL (green signal: vasculature). Scale bar: 100 µm. Insert: untreated ZFL. Right panel: 3D rendered orthogonal view of the left panel. Green signal: kdrl:EGFP. Red signal: DiI NPs. Scale bar: 100 µm. **c** Circulation factor (CF) is defined as the ratio between circulating (faint red) and bound (saturated red) NPs within the vasculature (green) of Tg(kdrl:EGFP) ZFL. Extravasation factor (EF) is the ratio between NP signal (red) inside and outside of the Tg(kdrl:EGFP) ZFL vasculature^58^. **d** Comparison of CF (left) and EF (right) between HEK293 and Huh7 nPMVs and native EVs, DOPC:PS, and DOPC liposomes 4 and 24 hpi in Tg(kdrl:EGFP) ZFL. Box plot: line: median, square: mean, box: lower and upper quartile, whisker: 1.5 interquartile range, filled square: outlier. *n* = 6 for HEK293 and Huh7 nPMVs and native EVs, *n* = 3 for DOPC:PS and DOPC liposomes. Levels of significance: *p ≤ 0.05, **p ≤ 0.01, ***p ≤ 0.001.

In vivo biodistribution of NPs was studied using ZFL as a vertebrate screening model^55^. For these experiments, we used the fish lines Tg(kdrl:EGFP)^56^ and Tg(mpeg1:Gal4:UAS:Kaede)^57^ both expressing green fluorescent proteins either in the vasculature or in macrophages, respectively (Fig. 5a). 48 h post fertilization of the ZFL, we injected DiI labeled HEK293 and Huh7 nPMVs and native EVs, DOPC:PS, and DOPC liposomes intravenously through the duct of Cuvier and imaged the red indicated tail region 4 h and 24 h post injection (hpi) using CLSM (Fig. 5b). Circulation and extravasation behavior of NPs was analyzed by semi-quantitative image analysis^58^. The fluorescence intensity of circulating NPs is a measure for their circulation behavior and described by the circulation factor (CF) (Fig. 5c). The extravasation factor (EF) defines the ratio between NPs located outside and inside the vasculature and is an indication for the extravasation behavior. We observed significant differences (*p* ≤ 0.001) in CF and EF at both, 4 hpi and 24 hpi between HEK293 and Huh7 nPMVs, DOPC:PS, and DOPC liposomes, which indicated a distinct biodistribution for each formulation. At 4 hpi, we observed CF_4h_ of 3.6–5.2 and EF_4h_ of 5.5–24.6 for HEK293 and Huh7 nPMVs and native EVs. These values did not significantly change after 24 h (CF_24h_ = 3.5–5.3, EF_24h_ = 3.9–12.9), being indicative of a short half-life in the circulation and extremely low extravasation (Fig. 5d). DOPC:PS liposomes had a significantly higher (*p* ≤ 0.001) CF and EF 4 and 24 hpi (CF_4h_ = 247 ± 29, EF_4h_ = 60 ± 17; CF_24h_ = 144 ± 23, EF_24h_ = 78 ± 31). DOPC liposomes showed the longest circulation time (CF_4h_ = 548 ± 38, CF_24h_ = 265 ± 33) and a strong extravasation (EF_4h_ = 128 ± 8, EF_24h_ = 190 ± 67) (*p* ≤ 0.001). This assessment was corroborated by analysis of 3D reconstructions of confocal image stacks, which allowed for a spatial localization of NPs relative to the vascular surface (Fig. 5b right panels). Positive or negative mean distance to surface values indicate that particles are mainly inside or outside the vasculature, respectively. The mean distance of HEK293 and Huh7 nPMVs and native EVs to the vascular surface was −3 to −4 µm, supporting the fast and efficient clearance observed by the low CF and EF values. For DOPC:PS liposomes, a mean distance to surface of −0.5 ± 1 µm was measured. This contrasted with DOPC liposomes devoid of PS, which showed a reduced interaction with scavenger endothelial cells and macrophages and a mean distance to the vascular surface of +3.0 ± 1 µm. In Tg(mpeg1:Gal4:UAS:Kaede) ZFL, we observed both empty and NP-filled macrophages for all NP formulations (Supplementary Fig. 24). Overall, a low level of colocalization of HEK293 and Huh7 nPMVs, native EVs, and DOPC liposomes with macrophages was calculated as indicated by the low Pearson value (*r* ≤ 0.3). DOPC:PS liposomes, however, showed a low to medium macrophage colocalization (*r* = 0.4)^59^.

## Discussion

The EV (i.e., nPMVs) preparation method described in this study (Fig. 1) is based on an initial observation made in 1979 that GPMVs can be produced by a stimulated membrane blebbing process^60^. Here, we refined this method to produce nano-sized GPMV-derived vesicles, nPMVs, which we systematically compared to native EVs and synthetic liposomal formulations.

In apoptotic cells, the asymmetric distribution of PS in the inner and outer leaflet of the cell membrane is lost^61^. Cell membrane blebbing and the production of GPMVs occurred in response to chemical stress-induced apoptosis. We observed binding of annexin V Alexa Fluor 488 to GPMVs, which indicated that GPMVs largely overlap with ApoBDs. The sizes of GPMVs were in line with previously reported sizes for ApoBDs (1–5 µm)^62^, further supporting the notion that GPMVs primarily comprise ApoBDs. We showed that GPMVs can be generated by chemical stressor (i.e., a combination of PFA and DTT^60,63^, diallyl disulfide^64^, and n-ethylmaleimide^65^) or osmotic buffer^66^. However, the highest yields were achieved with a combination of 25 mM PFA and 2 mM DTT followed by centrifugation at 100 × *g*. Within 1–6 h, a reproducible and high amount of GMPVs was obtained. Generally, GPMV production is limited to 6 h which results in ~24–36 GPMVs/cell. A previous study showed that higher PFA concentrations (250 mM) would further increase the yield^22^. However, we were interested in using conditions for optimized yield while keeping the chemical stressors at the lowest possible concentration to minimize cross-linking or breaking of disulfide bonds in proteins. Interactions of particles with biological systems are size-dependent^67^. For lipid-based drug formulations, a *D*_H_ between 80 and 150 nm is optimal^68^. Out of the three size reduction methods, only extrusion yielded formulations with a monodisperse and reproducible size distribution. Scale-up has been demonstrated by replacing a hand-held 1 mL extruder with a pressurized 100 mL extrusion device without the need for additional optimization.

After extrusion, the toxic chemical stressors (PFA and DTT) were removed by dialysis to avoid interfering with characterization assays (i.e., proteomics and lipidomics) and further use in in vitro/in vivo experiments. Non-dialyzed nPMVs significantly reduced the viability of Huh7 cells, whereas dialyzed nPMVs showed no changes in cell viability, confirming the successful removal of these toxic chemical stressors. As an alternative to dialysis, size exclusion chromatography could be used to rapidly remove these chemicals. With respect to GMP production of nPMVs and in view of future clinical use, it will be important to demonstrate the absence of the toxic chemical stressors PFA and DTT in the final preparation. Most guidelines state a short-term inhalation exposure limit to PFA of 2 ppm for 15 min. The compound is banned from use in cosmetics^69,70^ and no guidelines for parenteral use of PFA or DTT exist as these are not used as excipients in pharmaceutical products. However, DTT is poorly stable in biological environments and can easily be removed through dialysis or filtration^71,72^. It remains to be evaluated if alternative pharmaceutically compatible reducing agents (i.e., sodium thioglycolate, cysteine, reduced glutathione) could be used as substitutes^73^.

The question arises regarding the homogeneity and purity of our nPMV preparations. Compared to GPMVs, nPMVs have a more defined and uniform size distribution due to homogenization through extrusion. To further characterize possible heterogeneity of subpopulations, additional studies will be needed to analyze the cargo distribution and the protein and lipid composition of GPMVs or nPMVs at the single vesicle level such as by single vesicle imaging^74^ or flow cytometry of GPMVs^27,75^. A recent publication^76^ suggested that the ratio of particles per ug protein could be used as a measure for particle purity. According to this criterium our nPMV and native EV preparations were of high purity based on vesicular over non-vesicular protein contaminants. However, as discussed in the referenced publication, the method assumes that each vesicle has a comparable and constant quantity of protein, which is unlikely to be strictly true. In addition, the aspect of purity should also consider other types of contaminants such as chemical stressors, particle cargo, or aggregated materials. With respect to EV production, a mild and size-based purification method was applied (i.e., ultrafiltration and subsequent size exclusion chromatography). In contrast to ultracentrifugation-based approaches, co-isolation of non-vesicular protein particles, vesicle fusion, aggregation, and/or disruption can thus be reduced^12,26,74,77,78^.

Our analysis indicated an nPMV production rate of 1100-1400 nPMVs/cell/h and surpasses the native EVs (5-7 particles/cell/hour) and previously reported (60–170 particles/cell/h)^15^ production rates by more than two orders of magnitude. This exceptionally high nPMV production yield would allow to manufacture doses of 10^9^–10^12^ EVs, which are currently utilized in clinical trials^79^, within <6 hours by only using around 1.5 × 10^7^ donor cells (approx. one 15 cm cell culture dish or T-175 flask).

Our experimental findings are in good agreement with theoretical calculations assuming an average size of a mammalian donor cell of 15 μm, a mean GPMV size of 2 µm, and 120 nm large nPMVs. The surface of 56 spherical GMPVs would thus be equal to the surface of the donor cell. We found that our GPMVs carry up to 65% of the total membrane surface of their donor cells. Since up to 59% of the cell surface of our donor cells is recovered in the form of nPMVs, we conclude that GPMV secretion upon chemical stress is an extremely efficient process. We can therefore also support the observation of previous studies that stressing donor cells increases EV secretion^22–24^.

Stability experiments indicated that both nPMVs and native EVs, have excellent colloidal stability when stored at 4 °C for up to 1 month. However, a recent major stability/storage study on EVs showed that short-term storage (up to 8 days) in phosphate-buffered saline (PBS) led to decreased particle concentration and RNA amounts, whereas particle size and protein amount appeared unaffected^80^. The stability of nPMVs and native EVs might be further increased by PEGylation^81^, storage at −20 °C or −80 °C^80,82^, usage of optimized storage buffers (i.e., PBS including HEPES, albumin, and trehalose)^80^, lyophilization, or the addition of 0.05% NaN_3_ as an antibacterial agent^83^.

Our proteomic results indicated that nPMVs inherit proteins form their donor cells. Furthermore, nPMV proteins that did not overlap with the corresponding donor cells might exist in low quantities within the donor cells, making them undetectable in the extensive donor cell proteome. An additional explanation could be that the proteomes retrieved from the database do not entirely align with the proteome of our donor cell lines. By comparing proteomes of nPMVs derived from five different cell lines, we identified a set of common nPMV proteins but also donor cell line-specific proteins. Similar findings were previously described for other EVs^8^. The common nPMV proteins may serve as nPMV markers. Similarly, Kugeratski et al. detected a set of canonical proteins in exosomes irrespective of the donor cells^7^. These common nPMV proteins compromised several membrane proteins (i.e., CD proteins and integrins) with known relevance in cell surface interactions and cell uptake^84^. Most DAMPs are released upon cell damage and bind to Toll-like receptors of immune cells, which promotes the production of pro-inflammatory cytokines, chemokines, and IFN-I upon activation^85,86^. Some heat shock proteins, such as HSP60, HSP70, and HSP90, can be membrane-bound and can induce immune reactions. Furthermore, the nPMV proteome contains cell adhesion molecules and extracellular components such as those found in EVs, extracellular organelles, and extracellular exosomes confirming a pronounced plasma membrane protein enrichment, as previously described^7^. All together, these proteomic results support that nPMVs are equipped with a broad range of proteins involved in immune interactions and hence might be immunomodulatory. nPMVs of antigen-presenting cells could in this sense be used as cell-free cancer vaccines, as previously described for dendritic cell exosomes (dexosomes)^87^.

Considerable overlap was detected between the proteomes of HEK293 or Huh7 nPMVs and the corresponding native EVs. However, we documented fundamental differences in the proteomes of nPMVs and native EVs, which are largely consistent with their unlike cellular origin and preparation.

Interestingly, we did not detect CD63 in any nPMV preparations. There could be several explanations for this phenomenon: First, only a fraction of CD63 localizes to the plasma membrane as compared to intracellular compartments (endosome, MVBs, and lysosomes)^74,88–92^. Second, even in preparations that are enriched for exosomes, CD63 is typically not detected as highly abundant. In HEK293 EVs, for example, only 50% of all EVs are CD63 positive as assessed by single vesicle super-resolution imaging^74^. In addition, CD63 is one of the least abundant EV markers detected by LC-MS proteomics^74^. This could be due to either a low molecular density of CD63 per vesicle and/or technical limitations of LC-MS detection. Indeed, the poor ionization of the CD63 peptides might be caused by the high level of glycosylation of this marker.

Of all the bona fide exosome markers (i.e., Alix, TSG101, flotillin-1/2, LAMP1), we only detected Alix in nPMVs. This could have several reasons: First, Alix is also involved in apoptosis and shedding of the damaged cell membrane together with ALG-2^93^. Second, other studies have reported the presence of Alix in ectosomes^1,94^. Third, Alix is a cytosolic protein and thus could be transferred to GPMVs during cell blebbing and subsequently to nPMVs. Additionally, we detected several other markers for both, ApoBDs as well as ectosomes^28,62^. Taken together, our findings indicated that nPMVs comprise primarily ApoEVs, with little to no significant levels of exosomes. This notion is further supported by a previous study describing that EVs bearing CD9, CD81, BSG, and SLC3A2 with little CD63 bud mainly from the plasma membrane^28^. On the other hand, EVs bearing CD63 with little CD9 but including proteins from the late endosome (e.g., LAMP1, syntenin-1) derive from the internal compartments. The high whole proteome correlations expressed by the Pearson’s *r* values further showed that our nPMV preparation protocol yields nPMVs with highly reproducible proteome.

Lipidomic analysis showed an enrichment of PS, SM, phosphatidylcholine, and cholesterol esters in Huh7 nPMVs compared to their donor cells. Whereas, most EV studies showed enrichment of Chol, PS, SM, and glycosphingolipids in EVs derived from various cell lines (i.e., Huh7, PC-3, HepG2, dendritic cells, B-lymphocytes)^95,96^. These differences likely arose from the unlike cellular origin and preparation of nPMVs and native EVs^96^. We observed an almost four times higher PS content in nPMVs compared to donor cells, whereas most studies on EVs showed only a one- to three-fold enrichment. According to literature, PS contents in ApoBDs^62^ and exosomes^97,98^ can vary depending on the vesicle cellular origin or phenotype of the donor cells. However, for the biological properties, the location of PS in the membrane of NPs is more relevant than the overall PS content. PS exposed on the outer leaflet provides a potent “eat-me” signal for the TIM and TAM (Tyro3, Axl, and MerTK) receptors^99^. This presentation of PS is widely exploited by membrane-enveloped viruses (i.e., EBOLA, Dengue), and parasites^40–42^ as an essential co-factor for endocytic cell entry^43,44^. Using FCS to monitor annexin V binding, we showed that PS was exposed on the surface of nPMVs and native EVs^46^. This could support that our nPMVs, which were derived from GPMVs (ApoBDs), in fact primarily comprise ApoEVs. The presence of PS on the outer leaflet on exosomes and EVs, in general, is still controversially discussed. This may vary with the contribution of ApoBDs or ApoEVs in EV preparations as well as the cell types, since in particular subtypes of tumor cell-derived exosomes were reported to carry PS on the outer leaflet^42,97,98,100^. In healthy cells, proteins (i.e., flippases) actively flip PS from the outer to the inner leaflet of the cell. In NPs however, active flipping of PS is no longer possible. Upon storage, PS can thus naturally translocate to the outer leaflet until an equilibrium is reached. These two reasons could explain the binding of annexin V to native EVs as observed by FCS. In addition, we would like to note that our FCS results do not allow us to conclude on the density of surface exposed PS, as well as fraction of vesicles with exposed PS. This would require further experiments with single vesicle resolution. SM enrichment was observed for Huh7 nPMVs and native EVs, whereas phosphatidylinositol and phosphatidylethanolamine were depleted in both vesicles^95,96^. Cholesterol esters were enriched in nPMVs compared to donor cells, similar to previous reports for other ectosomes (i.e., microvesicles) but not for exosomes^96^. The absence of diglyceride and triglyceride in our nPMV preparations provided evidence that lipid droplets or other lipoparticles were either absent or present in negligible amounts. Of note, our lipidomic analysis did not cover Cholesterol and glycosphingolipids.

MTS assay results showed that dialyzed nPMVs and native EVs are non-toxic to Huh7 cells. Surprisingly, uptake experiments in Huh7 cells or THP-1 M0 macrophages using flow cytometry revealed no significant differences between the internalization of HEK293 and Huh7 nPMVs and native EVs and showed similar behavior for nPMVs across five cell types, including previously reported hallmarks of canonical exosome uptake routes involving filopodia recruitment and susceptibility to different uptake inhibitors^26^. In our experiments, cellular interactions of nPMVs and native EVs were solely dependent on the used target cells (i.e., Huh7 and THP-1 M0 macrophages). This is in contrast to the initial hypothesis that targeting/tropism of EVs can be modeled in vitro^101^. Consistent with more recent observations by many laboratories in the field, we also conclude that EV uptake efficiency in vitro is primarily determined by the recipient cell rather than the EV cell source, and that tissue tropism may need to be investigated in vivo. The similarity between nPMVs and native EVs could partially be explained by the observation that the lipid composition of NPs has a strong impact on their cellular interactions. This effect might supersede effects mediated by NP proteins (i.e., integrins, tetraspanins) and proteoglycans^44,102^. As described above, PS exposed on the surface of NPs serves as a potent “eat-me” signal and mediates cellular uptake^43^. Indeed, several lines of evidence support the notion that NP uptake may involve PS: First, in a series of control experiments with liposomes, DOPC:PS liposomes showed a more than 100-fold higher cellular uptake as compared to DOPC control liposomes in Huh7 cells. Second, we could demonstrate rapid cellular uptake and intracellular trafficking to the endo/lysosomal compartment for nPMVs, native EVs, and DOPC:PS liposomes, but not for DOPC liposomes devoid of PS. Third, blocking PS binding to cellular receptors using annexin V had a strong inhibitory effect on cellular endocytosis of nPMVs, native EVs, and DOPC:PS liposomes. Taken together, these data suggest that PS plays a role in cellular uptake of nPMVs, native EVs, and synthetic liposomes. In THP-1 M0 macrophages, however, an extremely low uptake of DOPC:PS liposomes was observed. Professional phagocytes (e.g., macrophages) recognize “eat-me” signals (e.g., PS) of apoptotic cells or vesicles through a set of receptors^99,103,104^. PS was the only “eat-me” signal in DOPC:PS liposomes and might thus not be enough to trigger engulfment by THP-1 M0 macrophages in this in vitro model.

nPMVs and native EVs were internalized via different energy-dependent endocytic pathways, as previously described for EVs, liposomes, and lipid nanoparticles^44,105^.

CLSM imaging of Huh7 and THP-1 M0 cells revealed that all NPs besides DOPC liposomes were internalized as single vesicles within minutes after addition, supporting the uptake trends observed by flow cytometry. Consistent with the canonical uptake and trafficking routes of CD63-GFP positive exosomes described previously^26^, live cell imaging and intracellular single particle trajectories showed that these NPs were shuttled in endosomes towards the endoplasmic reticulum and finally sorted to lysosomes, as observed by Lysotracker. Mean particle trafficking speeds of nPMVs and native EVs were significantly higher compared to DOPC:PS liposomes. All these results indicated that EVs, whether secreted naturally (i.e., native EVs) or in response to chemical stress (i.e., nPMVs) have superior cell interaction and intracellular processing compared to synthetic delivery vehicles—consistent with the recurring noting that their behavior is similar to viruses and other pathogens^26,40–42,106^.

Intravenously administered nPMVs and native EVs were rapidly sequestered mainly by scavenger endothelial cells in the caudal vein plexus possibly through scavenger receptors (i.e., stabilin)^107–109^ and dynamin-dependent endocytosis^110^ but also to lesser extent by tissue resident/patrolling macrophages. No uptake by other cell types was observed, this further highlights the specificity of these NPs to target cells. The fast clearance was moreover supported by the low CF and EF values observed for these NPs. It is interesting to note that identical interactions and observations were previously described for other EVs^110–112^. Comparative studies with PS containing lipid nanoparticles confirmed the important role of PS with respect to cellular uptake, tissue distribution, and extravasation^105^. Previous observations on the ZFL as an in vivo prediction tool indicated that clearance of NPs by scavenger endothelial cells and macrophages in ZFL will translate to accumulation of intravenously injected NPs in the liver and spleen of other in vivo models (i.e., rodents)^113,114^. Other studies in mice support the notion that intravenously injected exosomes are primarily sequestered by the liver, spleen, and kidney^115,116^. Thus, we speculate that nPMVs and native EVs would behave similarly in rodents. Overall, we showed that the composition of NPs largely influences the interactions with different cell types, circulation extravasation, and ultimately the biodistribution in vivo in ZFL^58^.

The distinct phenotypic and functional differences between ectosomes (e.g., ApoEVs, microvesicles) and native EVs and especially exosomes require a thorough characterization of biological consequences in recipient cells and organisms to instruct the choice of applications for nPMVs. Antigen-presenting ApoEVs were able to induce a higher cancer protection rate in tumor-challenged mice than EVs or microvesicles^117^. Of interest, apoptotic cell therapies have recently progressed into phase II clinical trials for the treatment of organ failure associated with sepsis and solid tumors^118–120^. The technology described in this study could serve as a surrogate for apoptotic cells. While this is beyond the scope of this study, follow-up work will be required to analyze the transcriptome and investigate potential applications for nPMVs as ApoEVs, such as vaccines, immunotherapies, drug delivery vehicles, and tissue regeneration (i.e., cardiovascular system, skin, bone, muscle, and kidney)^5,121^.

In the present work, we developed an EV preparation method yielding nPMVs and improved several limitations of existing EV preparation methods (i.e., efficiency, monodispersity, reproducibility): Our method surpasses the production rate and yield of conventional EV preparation methods by at least one to two orders of magnitude and offers the possibility to readily produce EVs with high size homogeneity and reproducibility (i.e., physico-chemical properties, proteome) in a shorter period of time (i.e., 6 h). A systematic comparison between nPMVs and native EVs from the same cells revealed biologically relevant differences in proteomes and lipidomes reminiscent of the unlike origin and preparation of nPMVs and native EVs. nPMVs are extruded and homogenized GPMVs (ApoBDs), which were produced by membrane blebbing of apoptotic cells, whereas native EVs comprise exosomes released through exocytosis from multivesicular bodies and plasma-derived nanovesicles actively secreted by healthy (non-apoptotic) cells. Additionally, our in vitro and in vivo studies evidenced that the lipid composition of NPs, such as the presence of PS on the outer leaflet of the NP membrane, highly influences particle-cell interactions but also the biodistribution in vivo. Ultimately, the EV preparation technology described in this study may therefore serve as a base for the development of therapeutic agents, which will either use nPMVs as a stand-alone product for immunotherapy or in combination with low molecular drugs or therapeutic biomolecules.

## Methods

### Preparation of GPMV

GPMVs were prepared using different cell lines. Cells were cultured as follows: A549 (ATCC, Manassas, VA), HEK293 EBNA (herein referred to as HEK293, ATCC), HepG2 (ATCC), and Huh7 (ATCC) cells were kept in Dulbecco’s Modified Eagle’s Medium with high glucose (Sigma-Aldrich, Buchs, Switzerland) supplemented with 10% fetal calf serum (Bioconcept, Allschwil, Switzerland), and 1% penicillin-streptomycin (Sigma-Aldrich). THP-1 monocytes (ATCC) were cultured in RPMI medium (Sigma-Aldrich) supplemented with 10% fetal calf serum, 1% penicillin-streptomycin, 50 µM 2-mercaptoethanol (Sigma-Aldrich), 1 mM sodium pyruvate (Bioconcept), and 10 mM HEPES pH 7.4 (Bioconcept). To differentiate TPH-1 cells from THP-1 M0 macrophages, the cells were incubated with 100 nM Phorbol-12-myristat-13-acetat (Sigma-Aldrich) for 48 h^122^. All cell lines were incubated routinely in a humidified CO_2_-incubator (5% CO_2_) at 37 °C and were confirmed to be free of mycoplasma.

For the production of GMPVs, cells were grown to 70% to 80% confluency on collagen I coated tissue culture plates (1.5 µg/cm^2^; Gibco, Thermo Fisher Scientific, Basel, Switzerland), washed with Dulbecco’s Phosphate-Buffered Saline (without CaCl_2_ and MgCl_2_; Sigma-Aldrich), and exposed for 6 h to freshly prepared GPMV buffer (150 µL/cm^2^; 10 mM HEPES, 150 mM NaCl, 2 mM CaCl_2_, pH 7.4) including chemical stressors (i.e., 25 mM PFA (Sigma-Aldrich), 2 mM DTT (Sigma-Aldrich)). Alternatively, the following chemical or osmotic stressors were used as indicated: 2 mM diallyl disulfide^64^, 2 mM n-ethylmaleimide^65^, or osmotic buffer (200 mM NaCl, 5 mM KCl, 0.5 mM MgCl_2_, 0.75 mM CaCl_2_, 100 mM bicine, pH 8.5)^66^. GPMV containing cell culture supernatant was gently collected and centrifuged (100 × *g*, 10 min, 4 °C) to pellet detached cells and large cell debris. Only the top 75% of the supernatant was collected to prevent contamination of GPMVs with cells and debris.

### Preparation of nPMVs

GPMVs samples with a maximal volume of 1 mL were extruded 13 times through polycarbonate filter membranes with pore sizes of 100 nm (Nucleopore; Whatman, North Bend, OH) or otherwise as indicated using a hand-held extruder (Avanti, Alabaster, AL). For larger volumes (up to 100 mL), a barrel extruder (Pevion Biotech, Bern, Switzerland; 600 psi N_2_, 13 extrusion runs, 100 nm filter membranes (Nucleopore; Whatman)) was used. Where indicated, 1:1000 dilution of 1 mM DiI in DMSO was added to the nPMV suspension to achieve a nominal concentration of 1 µM DiI and subsequently incubated in a thermomixer (Thermomixer comfort; Eppendorf) (30 min, 37 °C, 300 rpm). To remove free DiI, three washing steps with PBS using Amicon Ultra-4 filters (10 kDa MWCO, 20–30 min, 4000 × *g*, 4 °C) were performed. Alternatively, a 1:1000 dilution of 0.5–5 µM DiI in DMSO with nPMVs can be carried out before the extrusion process. In this case, no washing step is needed since the free dye is removed through the extrusion process. For some pilot experiments, microfluidics or sonication was used as an alternative to extrusion. Microfluidics was performed using a microfluidic mixing device (NanoAssemblr Benchtop, Presicion Nanosystems, Vancouver, Canada) with the indicated flow rates^123^. For sonication, we used a Branson Tip Sonifier 250 (Heinemann Labortechnik, Schwäbisch Gmünd, Germany) operated for 5 min with an output power of up to 120 W while cooling the sample in an ice-water bath.

After size homogenization, nPMVs were purified from PFA and DTT by dialysis (50 kDa MWCO, Spectra-Por 6 pre-wetted RC tubing; Spectrum Laboratories, Rancho Dominguez, CA) against a 1000-fold volume of GPMV buffer over 60 h with two changes of buffer. nPMVs were used immediately after dialysis. For higher concentrations, nPMVs were concentrated by centrifugation (4000 × *g*, 15–20 min, 4 °C) using Amicon Ultra-4 filters (10 kDa MWCO, Merck, Burlington, MA).

### Preparation of native EVs

HEK293 and Huh7 cells were cultivated in Dulbecco’s Modified Eagle’s Medium supplemented with 10% fetal bovine serum (Gibco), 1% GlutaMAX Supplement 100× (Gibco), and 1% antibiotic/antimycotic (100×, Gibco), and incubated at 37 °C, 5% CO_2_ and 90% relative humidity. For EV isolation from HEK293 or Huh7 cells, 2 × 10^7^ cells were seeded in T225 flasks or 4 × 10^7^ cells in 3-layered T-175 flasks (Falcon; Sigma-Aldrich) and were left to attach overnight. The cells were washed 1× with PBS and Opti-MEM supplemented with 1% antibiotic/antimycotic was added for conditioning. After 48 h of incubation, the conditioning medium was collected and centrifuged twice at 300 × *g* for 5 min and once at 3000 × *g* for 15 min at 4 °C. The conditioning medium was filtered through a 0.22 µm filter and was further concentrated 50–100× via ultrafiltration using 100 kDa MWCO Amicon filter (Merck) in a 200 mL Amicon Stirred Cell (Merck). After another 0.22 μm filtration step, the concentrate was further fractionated by size exclusion chromatography over a Superdex 20 Increase 10/30GL column (GE Healthcare, IL) on a Shimadzu LC-20AI FPLC instrument (Shimadzu, Korneuburg, Austria). The Shimadzu custom built FPLC system is mounted in a cold cabinet to ensure constant temperature of 4 °C and is equipped with a mobile phase degassing unit (Shimadzu DGU-20A3R), three liquid chromatography pumps, (Shimadzu Prominence LC-20Ai) a PDA detector (Shimadzu SPDM20A) and a fluorescence detector (Shimadzu RF-20A) with automated fraction collection (Shimadzu FRC-10A). The sample was eluted under isocratic conditions at 4 °C and 0.8 mL/min in PBS (8 g NaCl, 0.2 g KCl, 1.44 g Na_2_HPO_4_, 0.24 g KH_2_PO_4_ per L) adjusted to a pH of 7.4 and filtered through a 0.22 μm filter. Elution was monitored by 280 nm absorbance detection. Native EV-containing fractions were pooled and further concentrated via Amicon Ultra-4 filters (10 kDa MWCO, Merck). The samples were stored at 4 °C until further use. Where indicated, 1:1000 dilution of 1 mM DiI in DMSO was added to the native EV suspension to achieve a nominal concentration of 1 µM DiI and subsequently incubated in a thermomixer (Thermomixer comfort; Eppendorf) (30 min, 37 °C, 300 rpm). To remove free DiI, three washing steps with PBS using Amicon Ultra-4 filters (10 kDa MWCO, 20–30 min, 4000 × *g*, 4 °C) were performed.

### Preparation of liposomes

For control experiments, synthetic liposomes were formulated using a thin film hydration method^124^. In brief: for pure 1,2-dioleoyl-sn-glycero-3-phosphocholine (DOPC; Avanti) liposomes (PS-negative control) 2.5 µmol DOPC was dissolved in 500 µL of chloroform-methanol (2:1) in a round bottom flask. Similarly, 2.28 µmol DOPC was mixed with 0.25 µmol L-α-phosphatidylserine (herein referred to as PS; Avanti) to create DOPC:PS (PS-positive control) liposomes at a molar ratio of 9:1. The solvent was evaporated for 30 min at 70 °C using a Rotavapor A-134 (Büchi, Flawil, Switzerland). The dried lipid film was then immediately hydrated with 2 mL of GPMV buffer for 15 min by gentle stirring to reach a final lipid concentration of 1 mg/mL. The heterogeneous dispersion was extruded 13 times through 100 nm polycarbonate filter membranes (Nucleopore; Whatman) using a hand-held extruder (Avanti). After size homogenization, liposomes were dialyzed (50 kDa MWCO, Spectra-Por 6 pre-wetted RC tubing) against a 1000-fold volume of GPMV buffer over 60 h with two buffer changes. Freshly prepared liposomes were used for all experiments and stored at 4 °C.

### Physico-chemical characterization of vesicles

GPMV concentration was determined by visual counting using a light CKX 41 microscope (Olympus, Tokyo, Japan) in combination with a Neubauer Improved hemocytometer. The size of GPMVs was assessed by flow cytometry (FACSCanto II RUO flow cytometer; BD Biosciences, San Jose, CA) using calibration beads with sizes between 2 and 9 μm (Spherotech, Lake Forest, IL) and BD FACS DIVA Software V7. Annexin V Alexa Fluor 488 Ready Flow conjugate was used to detect apoptotic nature of GPMV by adding a one drop to the GPMV suspension and subsequent flow cytometry analysis (exc. 488 nm, em.: fluorescence channel FL1 (505 nm LP–530/15 nm). Data were analyzed using FlowJo V9 software (TreeStart, Ashland, OR).

*D*_H_, PDI, and ζ potential of nPMVs, native EVs, and liposomes were determined using the Zetasizer Ultra dynamic light scattering system (Malvern Panalytical, Volketswil, Switzerland) using the ZS Xplorer software V2.3.0.62 (Malvern Panalytical) according to standard protocols provided by the manufacturer. Size and size distribution were analyzed using the multiple narrow mode model. For ζ potential measurements, samples were diluted 1:10 with sterile filtered 5% glucose and 20 mM HEPES (pH 7.4) in H_2_O to reduce the free ion concentration. ζ potential was assessed in three separate runs using the Smoluchowski equation and automated recording settings.

### NTA

NTA of NPs was performed using a NanoSight NS 300 instrument (NanoSight Ltd, Salisbury, United Kingdom) equipped with a 488 nm laser, sCMOS camera, and a flow cell. The samples were diluted with buffer in a way that 75–100 particles were visible in the field of view, according to the manufacturer’s user manual. Then, diluted samples were applied to the viewing chamber using a syringe pump with a continuous flow of 100 µL/min while recording three videos of 60 s with 25 frames/sec at RT for each measurement. Camera and analysis settings were kept constant between samples. The NTA software V3.4 (NanoSight) was used to analyze the movement of NPs based on single particle tracking on a frame-by-frame basis (Brownian motion) in order to obtain their size distribution, mean/median *D*_H_, standard deviation, and particle concentration in solution.

### Cryo-TEM analysis and tomography

For cryo-TEM analysis, nPMVs and native EVs were concentrated (≥10^12^ particles/mL) using Amicon Ultra-4 filters (10 kDa MWCO, 20-30 min, 4000 × *g*, 4 °C, Merck). Samples were adsorbed onto a carbon-coated grid (Lacey, Ted Pella, CA) and vitrified by rapid transfer into liquid ethane using a Leica GP plunger (Leica, Wetzlar, Germany). Frozen grids were transferred into a Talos electron microscope (FEI, Hillsboro, OR) using a Gatan 626 cryo-holder. Electron micrographs were recorded at an accelerating voltage of 200 kV and a nominal magnification of ×73,000, using a low-dose system (20 e^−^/Å^2^) and keeping the sample at low temperature.

For cryo-TEM tomography, samples were premixed with gold fiducials (10 nm) and adsorbed onto carbon-coated grids as described above. Tilt series were acquired from −60° to +60° in 3° steps in consecutive order. The total dose received by 61 images corresponds to ~200 e^−^/Å^2^. Segmentation, generation of a nPMV density map, and sample visualization were performed^125^. The final rendering of the nPMV model was created with Blender V3.3 (Blender Foundation, Amsterdam, Netherlands).

### Fluorescence correlation spectroscopy (FCS)

FCS was used to determine particle concentration, brightness (counts per molecule) and *D*_H_ of DiI-labeled NPs. Measurements were performed with an Olympus IX73 inverted microscope equipped with a water immersion superapochromat objective (×60 UplanSApo (1.2 NA); Olympus), a pinhole of 100 µm, and diode lasers operated at 40 MHz.

For measurements of DiI labeled NPs, 10 nM Sulforhodamine B (*D* = 425 μm/s^2^ at 298 K; Invitrogen) dissolved in GPMV buffer was used to calibrate the confocal volume (exc. channel 530 nm). Intensity fluctuations were recorded over 180 s at 40 μm distance from the coverslip with a correlation integration time of 2 s. Emitted photons were detected with a SPAD detector (SPCM-AQRH-14-TR; Excelitas Technologies, Kehlheim, Germany) using a 586/75 nm BP filter for DiI. To calculate particle concentration, brightness, and size, experimental autocorrelation curves of NPs were fitted using a one-component pure diffusion law according to the Stokes-Einstein equation^126^ in the SymPhoTime64 V2.6 software (Picoquant, Berlin, Germany). For in vitro cell interaction studies, live cell imaging, and in vivo ZFL studies, DiI-labeled NPs were normalized to indicated concentrations of 5 × 10^10^ or 2 × 10^12^ particles/mL based on FCS measurements.

To confirm PS localization on the surface of NPs, annexin V Alexa Fluor 488 conjugate (Invitrogen) was added at 20 nM to the NPs and incubated at 4 °C for 30 min. Measurements were performed as described above using Atto 488 carboxylic acid (*D* = 400 μm/s^2^ at 298 K; Atto-Tech, Siegen, Germany) at 10 nM in GPMV buffer to calibrate the confocal volume of exc. channel 481 nm. Emitted photons were detected with a SPAD detector (SPCM-AQRH-14-TR; Excelitas Technologies) using a 512/25 nm BP filter in the emission path.

### Protein concentration measurements

Protein concentrations (absorbance at 280 nm and 1 A = 1 mg/mL) of nPMVs and native EVs were measured with a DeNovix DS-11+ spectrophotometer (DeNovix Inc., Wilmington, NC) GPMV buffer or PBS was used as blank.

### Proteomics

Two proteomic studies were performed. First, A549, HEK293, HepG2, Huh7, and THP-1 nPMV proteomes were compared (herein referred to as study 1). Second, proteomes of HEK293 and Huh7 nPMVs and native EVs were compared (herein referred to as study 2). The proteomes of HEK293^127^ and Huh7 cells^128^ were retrieved from PRoteomics IDEntification Database (PRIDE)^129^.

#### Sample preparation for MS-based proteome analysis

For both studies, NPs were reconstituted in buffer containing 5% sodium dodecyl sulfate (Merck), 100 mM tetraethylammonium bromide (Merck), and 10 mM tris(2-carboxyethyl)phosphine (Merck), and lysed by heating (95 °C, 10 min) and subsequent treatment by sonication in a BioRuptor (Diagenode, San Diego, CA) at 10 cycles (30 s on, 30 s off) at 4 °C for 10 min. Carbamidomethylation of cysteines was performed by addition of 20 mM iodoacetamide at 25 °C in the dark for 30 min. Samples were subjected to S-Trap-based digestion and purification according to the manufacturers procedure^130^ using 1 μg trypsin per sample on the S-Trap column. Eluates were dried in a vacuum concentrator and stored at −20 °C until further use. Dried peptides were dissolved in 0.1% aqueous formic acid solution at a concentration of 0.1 mg/mL prior to injection into the mass spectrometer.

#### Mass spectrometry analysis

0.25 μg (study 1) or 0.15 μg (study 2) of total peptides were subjected to either a LC-MS analysis using Q Exactive Plus Mass Spectrometer fitted with an EASY-nLC 1000 (Thermo Fisher Scientific) for the study 1 or a Orbitrap Fusion Lumos Mass Spectrometer fitted with an EASY-nLC 1200 (Thermo Fisher Scientific) for the study 2, both equipped with a custom-made column heater set to 60 °C. Peptides were resolved using a RP-HPLC column (study 1: 75 μm × 30 cm, study 2: 75 μm × 36 cm) packed in-house with C18 resin (ReproSil-Pur C18–AQ, 1.9 μm resin; Dr. Maisch GmbH, Ammerbuch, Germany) at a flow rate of 0.2 μL/min. The following gradients were used for peptide separation: Study 1: 5% B to 15% B over 10 min to 30% B over 60 min to 45% B over 20 min to 95% B over 2 min followed by 18 min at 95% B; Study 2: 5% B to 12% B over 5 min to 35% B over 40 min to 50% B over 15 min to 95% B over 2 min followed by 18 min at 95% B. Buffer A was composed of 0.1% formic acid in water and buffer B was composed of 80% acetonitrile, 0.1% formic acid in water. For study 1, the mass spectrometer was operated in DDA mode with a total cycle time of ~1 sec. Each MS1 scan was followed by high-collision-dissociation of the 20 most abundant precursor ions with dynamic exclusion set to 30 sec. For MS1, 3 × 10^6^ ions were accumulated in the Orbitrap over a maximum time of 250 ms and scanned at a resolution of 140,000 FWHM (at 200 m/z). MS2 scans were acquired at a target setting of 1 × 10^5^ ions, maximum accumulation time of 50 ms, and a resolution of 17,500 FWHM (at 200 m/z). Only peptides with charge state 2–5 were included in the analysis. The normalized collision energy was set to 27%, the mass isolation window was set to 1.4 m/z, and one microscan was acquired for each spectrum. For study 2, the Orbitrap Lumos mass spectrometer was operated in DDA mode with a cycle time of 3 sec between master scans. Each master scan was acquired in the Orbitrap at a resolution of 120,000 FWHM (at 200 m/z) and a scan range from 375 to 1600 m/z followed by MS2 scans of the most intense precursors in the linear ion trap at rapid scan rate with isolation width of the quadrupole set to 1.4 m/z. Maximum ion injection time was set to 50 ms (MS1) and 35 ms (MS2) with an AGC target set to 10^6^ and 10^4^, respectively. Only peptides with charge state 2–5 were included in the analysis. Monoisotopic precursor selection was set to peptide, and the intensity threshold was set to 10^4^. Peptides were fragmented by higher-energy collisional dissociation with collision energy set to 35%, and one microscan was acquired for each spectrum. The dynamic exclusion duration was set to 30 sec.

#### Protein identification and quantification

Raw-files were either searched using MaxQuant V1.6.14.0 (Max Plank Institute, Munich Germany) for study 1 or MSFragger^131^ V3.5 (Nesvizhskii lab, Ann Harbor, MI) using the FragPipe interface (Nesvilab/FragPipe, 2022) V18.0 and Philosopher V4.3.0 for study 2 against a decoy database containing normal and reverse sequences of the *human* UniProt proteome version of 2020-04-17 (study 1) or 2022-02-22 (study 2) and commonly observed contaminants (in total 41,484 sequences). The following search criteria were used for both studies: full tryptic specificity was required (cleavage after lysine or arginine residues, unless followed by proline); two missed cleavages were allowed; carbamidomethylation (C) was set as fixed modification; oxidation (M) and Acetyl (Protein N-term) was applied as variable modification. Match between runs option was disabled. The database search results were filtered using the ion score to set the false discovery rate to 1% on the peptide and protein level, respectively, based on the number of reverse protein sequence hits in the datasets. For study 1, quantitative analysis results from label-free quantification were normalized and statically analyzed using the DEP R package V1.2.0 (Bioconductor) to obtain differentially expressed protein abundances^132^. For study 2, qualitative overview of protein database search results was presented by Scaffold V5.2.0 (Proteome Software, Portland, OR) was used.

### Lipidomics

#### Lipid extraction for mass spectrometry lipidomics

Mass spectrometry-based lipid analysis of nPMVs (≥0.6 mg/mL) or cultured cells (3 × 10^6^ cells/mL) was performed by Lipotype GmbH (Dresden, Germany)^133^. Lipids were extracted using a chloroform/methanol procedure^134^. Samples were spiked with internal lipid standard mixture containing: diacylglycerol 17:0/17:0, phosphatidic acid 17:0/17:0, phosphatidylcholine 17:0/17:0, phosphatidylethanolamine 17:0/17:0, phosphatidylglycerol 17:0/17:0, phosphatidylinositol 16:0/16:0, PS 17:0/17:0, cholesterol ester 20:0, SM 18:1;2/12:0;0 and triacylglycerol 17:0/17:0/17:0. After extraction, the organic phase was transferred to an infusion plate and dried in a speed vacuum concentrator. The dry extract was re-suspended in 7.5 mM ammonium acetate in chloroform/methanol/propanol (1:2:4, v/v/v) and in 33% ethanol solution of methylamine in chloroform/methanol (0.003:5:1; v/v/v). All liquid handling steps were performed using Hamilton Robotics STARlet robotic platform with the Anti Droplet Control feature for organic solvents pipetting.

#### MS data acquisition

Samples were analyzed by direct infusion on a QExactive mass spectrometer (Thermo Fisher Scientific) equipped with a TriVersa NanoMate ion source (Advion Biosciences, Harlow, UK). Samples were analyzed in both positive and negative ion modes with a resolution of Rm/z = 200 = 280,000 for MS and Rm/z = 200 = 17,500 for MS-MS experiments, in a single acquisition. MS-MS was triggered by an inclusion list encompassing corresponding MS mass ranges scanned in 1 Da increments^135^. Both MS and MS-MS data were combined to monitor cholesterol ester, diacylglycerol, and triacylglycerol ions as ammonium adducts; PC as acetate adduct; and phosphatidic acid, phosphatidylethanolamine, phosphatidylglycerol, phosphatidylinositol, and PS as deprotonated anions. MS only was used to monitor SM as acetate adduct.

#### Data analysis and post-processing

Data were analyzed with in-house developed lipid identification software based on LipidXplorer^134,136^. Data post-processing and normalization were performed using an in-house developed data management system. Only lipid identifications with a signal-to-noise ratio >5, and a signal intensity five-fold higher than in corresponding blank samples were considered for further data analysis.

### MTS cell viability assay

Cell viability was measured using the MTS Assay Kit (Cell Proliferation, Colorimetric, ab197010). A working solution containing 2 mg/mL MTS (Sigma-Aldrich) and 0.21 mg/mL phenazine ethosulfate (Sigma-Aldrich) in Dulbecco’s phosphate-buffered saline was prepared^137^. For the cell viability measurement, 3.3 × 10^3^ Huh7 cells were plated on rat tail collagen I (4.5 µg/cm^2^; Gibco) coated 96 well plates and allowed to adhere for 24 h. NPs (5 × 10^10^ particles/mL, measured and normalized by FCS) were added to achieve a final concentration of 1 × 10^9^ particles/mL and incubated with Huh7 cells for 24 h. Then, 20 μL of the MTS/PES working solution were added. After 1 h, the formation of formazan dye was quantified by measuring absorbance at 490 nm (SpectraMax M2e, Molecular Devices, CA). Untreated Huh7 cells were used as reference (100% viability) and terfenadine (1–50 nM; Sigma-Aldrich) treated cells as a positive control (reduced cell viability).

### In vitro cell interaction and flow cytometry

Cellular interactions of NPs with cell lines were studied using flow cytometry. 1 × 10^5^ Huh7 or THP-1 M0 cells/well were plated on rat tail collagen I (4.5 µg/cm^2^, Gibco) coated 24 well plates and allowed to adhere for 24 h. The next day, DiI-labeled NPs (5 × 10^10^ particles/mL, measured and normalized by FCS) were added to reach a final concentration of 1 × 10^9^ particles/mL. Cells were further incubated, washed three times with PBS, detached at indicated time points with 0.25% trypsin-EDTA (Gibco), and analyzed using flow cytometry (FACS Canto II; BD Biosciences). Excitation wavelength was 561 nm and fluorescence of the detached cells (DiI signal) was detected using fluorescent channel FL5 (586/15 nm) (BD FACS DIVA Software V7). Data were analyzed using FlowJo V10.8.1 software (TreeStart), see Supplementary Fig. 18 for gating strategy. Median RFU of 10^4^ cells were analyzed to quantify the extent of cellular interactions of NPs with cells. To allow for a direct comparison between nPMVs, native EVs, and liposomes, median cellular RFU values were normalized according to the brightness of each NP, as determined by FCS analysis, and thus, represented as normalized cell RFU values.

To identify cellular uptake pathways/mechanisms, uptake studies in presence and absence of specific inhibitors were carried out. The following inhibitors were added 2 h before addition of DiI labeled NPs (5 × 10^10^ particles/mL, measured and normalized by FCS) to the cells: Cytochalasin B (10 μg/mL) and colchicine (10 μg/mL; Sigma-Aldrich). Chlorpromazine (10 μg/mL; Sigma-Aldrich), polyinosinic acid (10 μg/mL; Sigma-Aldrich), Rabbit anti-human-TIM-1 antibody (7.5 μg/mL; Invitrogen), nystatin (50 μg/mL; Sigma-Aldrich), or NaN_3_ (0.1% v/v and 1% v/v; Sigma-Aldrich) were preincubated with cells for 30 min. After 4 h incubation with NPs, cells were washed three times with PBS, detached, and analyzed by flow cytometry as described above. The change in NP uptake (%) is calculated as follows:

$${Uptake}\,{change}\,\left[ \% \right]=100* \frac{{{Cell}\,{RFU}}_{{inhibitor}\,{presence}}}{{{Cell}\,{RFU}}_{{inhibitor}\,{absence}}}-100$$

### Live cell imaging

CLSM was performed using an Olympus FV-3000 inverted microscope equipped with an oil immersion objective (×60 UPlanFLN (1.4 NA); Olympus). Laser and detection line settings were kept constant for the channels of interest. For live cell imaging, Huh7 cells and TPH-1 M0 macrophages were cultured for 24 h on collagen I coated (4.5 µg/cm^2^, Gibco) microscopy microslides (Ibidi GmbH, Gräfelfing, Germany). The stage and the incubator of the microscope were preheated to 37 °C and cells were maintained in a CO_2_ enriched (5%) and humidified atmosphere. Huh7 and THP-1 M0 cells were incubated with DiI labeled NPs (5 × 10^10^ particles/mL in complete cell culture medium) and directly imaged for 30 min using sequential scanning mode. Thereafter, the cells were stained with the following compartment-specific fluorescent cell markers as indicated and reimaged as Z-stacks: Chol-PEG-FITC (10 µM, 1 min incubation, cell membrane; Nanocs, Boston, MA); Hoechst 33342 (2.5 μg/mL, 4 min incubation, nucleus; Invitrogen); LysoTracker Green DND-26 (final dilution 1:1000, 5 min incubation, lysosomes; Invitrogen); CellTracker Deep Red (5 μM, 5–10 min incubation, cytosol cell staining; Invitrogen). Images were analyzed and processed (Z-projections at max. intensity) and orthogonal views) with Fiji/ImageJ software V1.53 q. Single particle trajectories and speeds of *n* ≥ 25 NPs in a total of *n* ≥ 3 cells were analyzed using the tracking/statistic plugin in Imaris software V9.9.0 (Bitplane, Belfast, UK) with the following settings: spot detection size: 0.5 µm, tracking parameter: autoregressive motion, max. distance: 10 µm, max. gap: 3 and track duration: 120 s.

### In vivo biodistribution studies in ZFL

All procedures on live ZFL (*Danio rerio*) were carried out according to the Swiss animal welfare regulations. The zebrafish lines Tg(kdrl:EGFP)^56^ and Tg(mpeg1:Gal4:UAS:Kaede) were kindly provided by Prof. M. Affolter and Dr. H Belting (Biozentrum, University of Basel, Switzerland). ZFL were maintained under standard conditions in zebrafish E2 culture medium at 28 °C^55^. To suppress melanogenesis, 30 μg/mL 1-phenyl 2-thiourea (Sigma-Aldrich) was added to the culture media 0, 24, and 48 h post fertilization. At 48 h post fertilization, 5 nL of nPMVs, native EVs, or liposomes (2 × 10^12^ particles/mL, measured and normalized by FCS) were injected intravenously into the duct of Cuvier. NP distribution within the tail region of the ZFL was visualized 4 h and 24 h post injection (hpi) by CLSM analysis (inverted Olympus FV-3000 microscope, ×20 UPlanSApo (0.75 NA); Olympus) acquiring Z-stacks (step size: 2 µm). Images were analyzed and processed with Fiji/ImageJ software V1.53 q. Semi-quantitative computer was performed to determine the CF and the EF^58^. CF values >200 are a characteristic of NPs with an extended circulation time. An EF > 270 is indicative of extensive extravasation as opposed to retention of NPs within the vasculature (EF < 70). The Pearson’s coefficients were obtained by analyzing the maximum intensity projections of equal heights of z-stacks with the JACoP plugin^138^ for the Fiji/ImageJ software^139^ V1.53 q. 3D rendered orthogonal views of Tg(kdrl:EGFP) ZFL were rendered by Imaris software. The vasculature (kdrl:EGFP channel) was simulated as a surface with the following settings: surface detail: 1.2 µm, threshold: 100, filter threshold: 10. NPs were replaced by spheres with 1 µm diameter: estimated spot detection diameter: 2 µm, filter type: quality with threshold value of 12.

### Statistics and reproducibility

Statistical analysis was performed using a two-way ANOVA with Bonferroni test including interactions between factors or by two-sample *t* test. Software used for statistical analysis and data representation was OriginPro v2022 (OriginLab, Northampton, MA). Levels of significance: *p ≤ 0.05, **p ≤ 0.01, ***p ≤ 0.001.

### Reporting summary

Further information on research design is available in the Nature Portfolio Reporting Summary linked to this article.

## Supplementary information

Supplementary Information

Reporting summary

## Data Availability

Raw data can be retrieved from the public data repository Zenodo (https://zenodo.org/record/7849411)^140^. Raw proteomics data can be retrieved from the MassIVE database (dataset: MSV000091730)^141^.
