# Supplementary material for: High efficiency preparation of monodisperse plasma membrane derived extracellular vesicles for therapeutic applications
Source: Commun Biol. 2023 May 3;6:478. doi: 10.1038/s42003-023-04859-2 (PMC10156699; doi:10.1038/s42003-023-04859-2)
Supplement: Supplementary file 1 — Supplementary Information [file 42003_2023_4859_MOESM1_ESM.pdf]

High efficiency preparation of monodisperse plasma membrane derived extracellular vesicles for therapeutic applications

Claudio L. Alter[1,2,3], Pascal Detampel[1], Roman B. Schefer[1], Claudia Lotter[1], Patrick Hauswirth[1], Ramya D. Puligilla[1], Vera J. Weibel[1], Susanne H. Schenk[1], Wolf Heusermann[4], Melanie Schürz[5], Nicole Meisner-Kober[5], Cornelia Palivan[3], Tomaž Einfalt*[1], Jörg Huwyler*[1]

[1] Department of Pharmaceutical Technology, University of Basel, Klingelbergstrasse 50, 4056 Basel, Switzerland

[2] Swiss Nanoscience Institute, University of Basel, Klingelbergstrasse 82, 4056 Basel, Switzerland

[3] Department of Chemistry, University of Basel, Mattenstrasse 24a, BPR 1096, 4058 Basel, Switzerland

[4] Imaging Core Facility, University of Basel, Spitalstrasse 41, 4056 Basel, Switzerland

[5] Department of Biosciences & Medical Biology, University of Salzburg, Hellbrunnerstrasse 34, 5020 Salzburg, Austria

*both authors contributed equally

Author for correspondence:
Jörg Huwyler, PhD
Professor of Pharmaceutical Technology
University of Basel, Department of Pharmaceutical Sciences
Klingelbergstrasse 50
CH-4056 Basel, Switzerland
joerg.huwyler@unibas.ch

Supplementary Figures

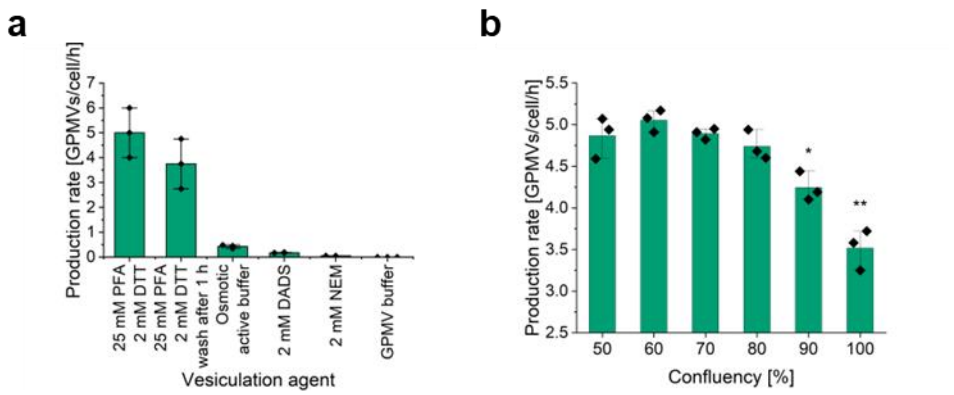

Supplementary Figure 1: HEK293 GPMV production rate of different chemical stressors and exposure times and as function of the cell confluency. a: GPMV production rate of HEK293 cells treated with different vesiculation agents was compared to our baseline (25 mM paraformaldehyde (PFA) and 2 mM dithiothreitol (DTT) for 6 h). Similar production rates were observed for the other tested cell lines (A549, HEK293, HepG2, and THP-1 M0). The other chemical stressors or the osmotic buffer were less efficient. Of note, GPMV formation is generally limited to 6 h and thereafter yields will not increase notably. **b:** GPMV production rate as function of donor cells confluency. Constant production rates were observed until cells reached a confluency of 70-80%. Values are means ± SD, squares: data points, n=3.

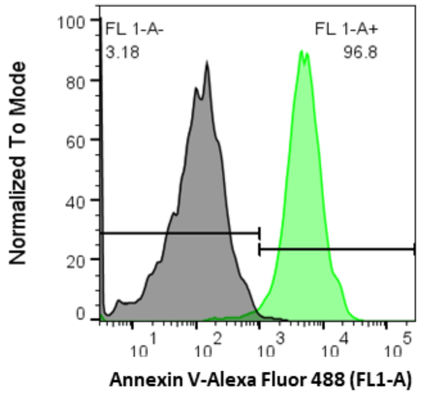

Supplementary Figure 2: Flow cytometry analysis of HEK293 GPMVs after incubation with annexin V Alexa Fluor 488. Representative HEK293 GPMVs were incubated with

phosphatidylserine (PS) binding annexin V Alexa Fluor 488 and analyzed by flow cytometry to test if GPMVs comprise apoptotic bodies (ApoBDs). Around 96.8% of GPMVs were annexin V Alexa Fluor 488 positive (FL 1-A+). Comparable results were observed for A549, HepG2, Huh7, and THP-1 M0 GPMVs. Black: GPMVs. Green: HEK293 GPMVs after incubation with annexin V Alexa Fluor 488.

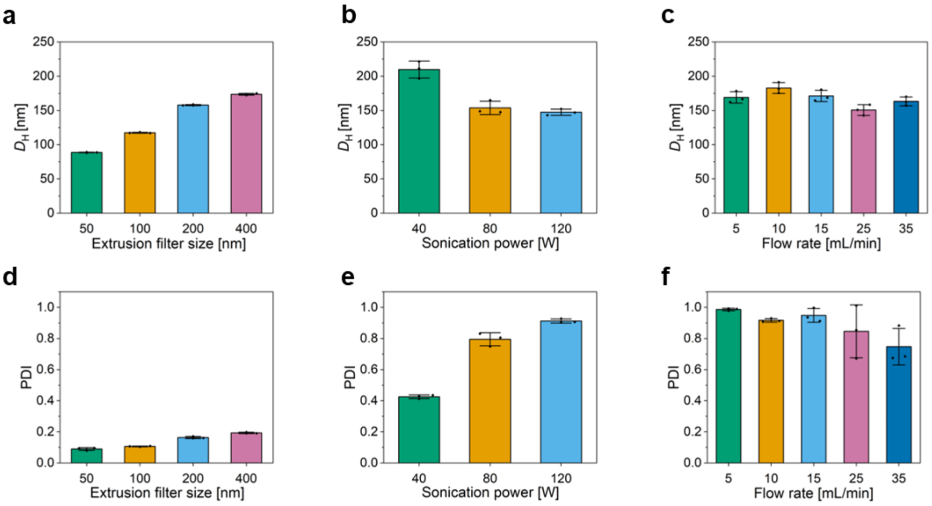

Supplementary Figure 3: Size homogenizations methods used to produce nPMVs from GPMVs. a, d: D_H (**a**) and PDI (**d**) of GPMVs through filter membranes of 50 (green), 100 (orange), 200 (blue), or 400 (pink) nm pore size were compared. Compared to microfluidic mixing and sonication, extrusion through filter membranes provided a monodisperse (PDI < 0.2) nPMV solutions as measured by DLS. **b, e:** D_H (**b**) and PDI (**e**) of nPMVs produced by sonication with an output power of 50 (green), 100 (orange), or 200 (blue) watts. **c, f:** D_H (**c**) and PDI (**f**) of nPMVs produced by microfluidic mixing at flow rates of 5 (green), 10 (orange), 15 (blue), 25 (pink), or 35 (dark blue) mL/min. Values are means ± SD, squares: data points, n=3.

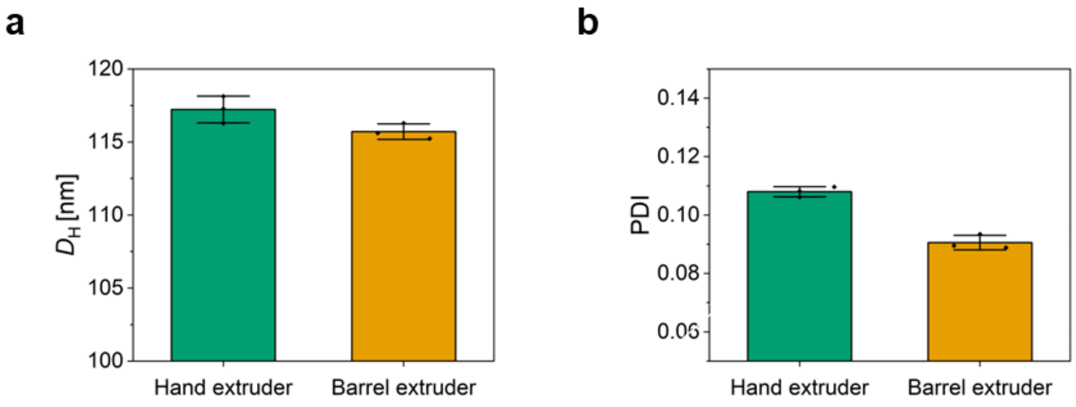

Supplementary Figure 4: D_H and PDI comparison of nPMVs homogenized by hand or barrel extruder. No significant D_H (**a**) or PDI (**b**) differences were observed between hand (green) and barrel (orange) extruder. Values are means ± SD, squares: data points, n=3.

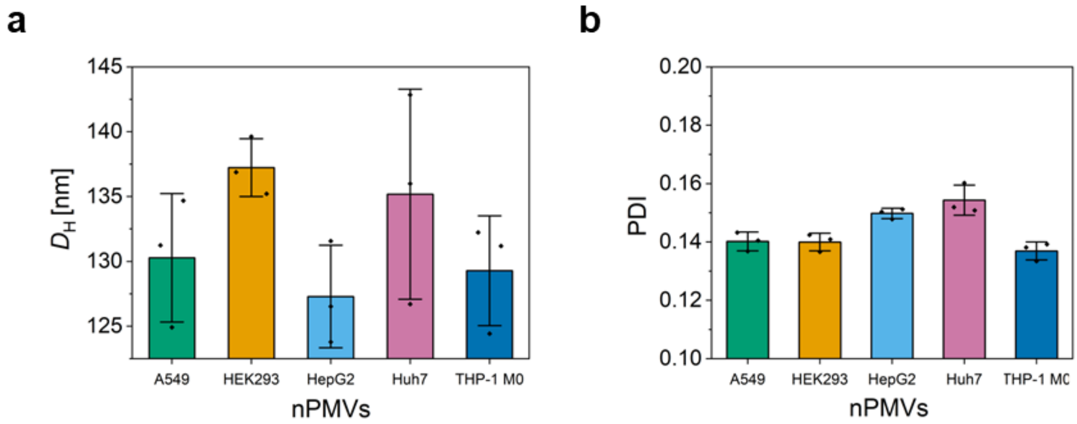

Supplementary Figure 5: Physico-chemical characterization of nPMVs derived from various cell lines. Comparison of D_H (**a**) and PDI (**b**) of A549 (green), HEK293 (orange), HepG2 (blue), Huh7 (pink), and THP-1 M0 (dark blue) nPMVs. No significant differences were observed. Values are means ± SD, squares: data points, n=3.

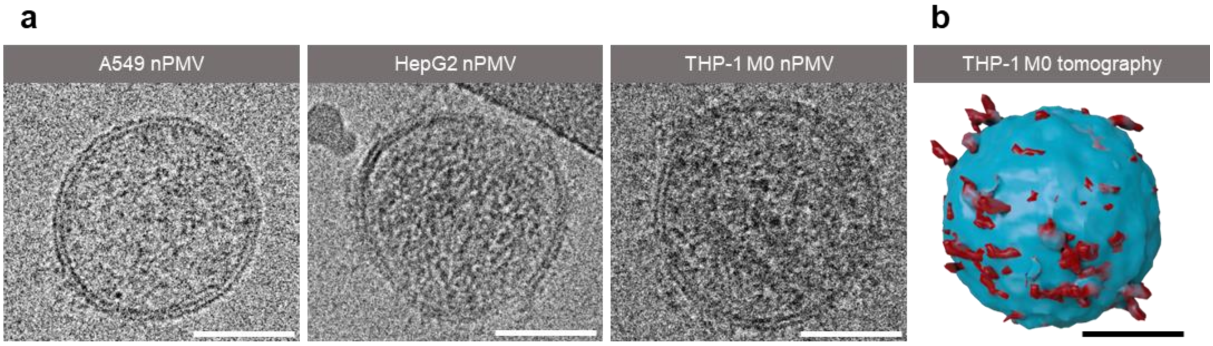

Supplementary Figure 6: Cryo-TEM images and tomography of selected nPMVs. a: A549, HepG2, and THP-1 M0 nPMVs were imaged using cryo-TEM. Scale bar: 50 nm. **b:** 3D reconstruction (tomography) of the THP-1 M0 cryo-TEM image in **a**. Scale bar: 50 nm. Blue: nPMVs membrane. Red: Membrane protruding proteins.

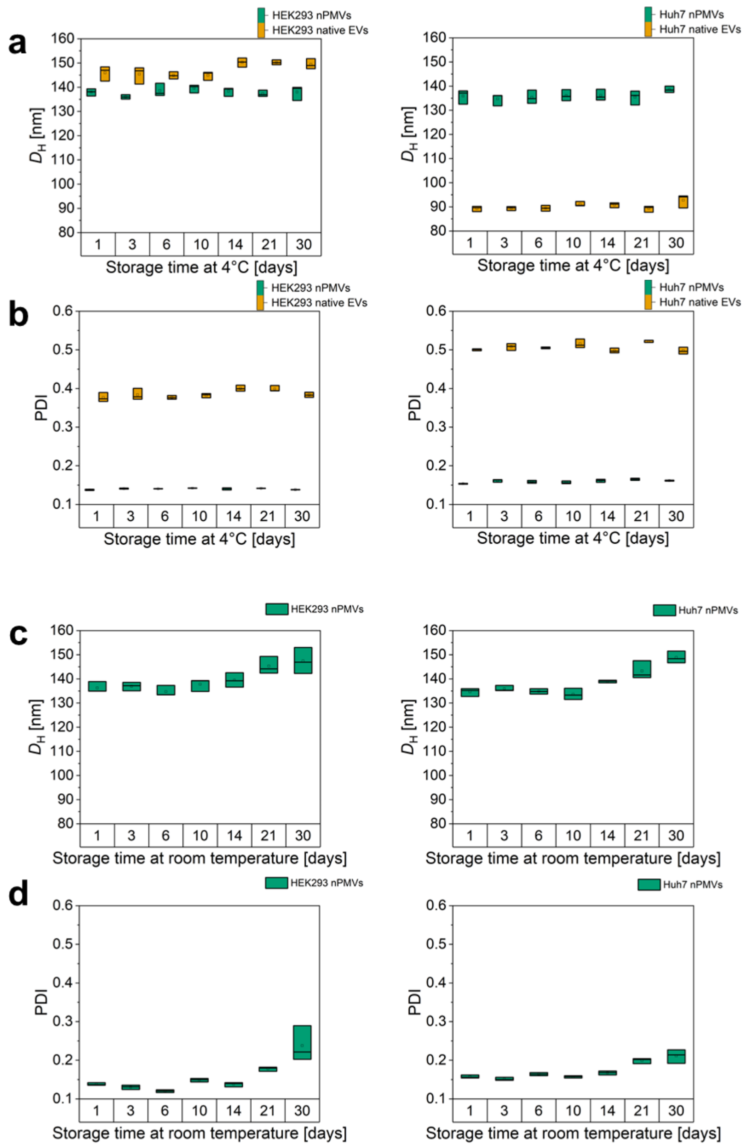

Supplementary Figure 7: Colloidal stability of HEK293 and Huh7 nPMVs and native EVs upon storage. a, b: D_H (**a**) and PDI (**b**) of HEK293 nPMVs and native EVs (left) or Huh7 nPMVs and native EVs (right) when stored at 4 °C and analyzed after indicated storage time. **c, d:** D_H (**c**) and PDI (**d**) of HEK293 nPMVs and native EVs (left) or Huh7 nPMVs and native EVs (right) when stored at RT and analyzed after indicated storage time. Green indicates nPMVs. Orange indicates native EVs. Box plot: line: median, square: mean, box: lower and upper quartile, whisker: 1.5 interquartile range, filled square: outlier, n=3 measurements.

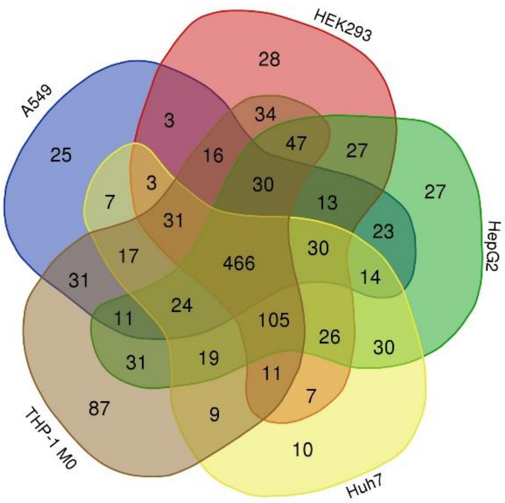

Supplementary Figure 8: Venn diagram of five different nPMV types. 466 common proteins were detected between the proteomes of A549 (blue), HEK293 (red), HepG2 (green), Huh7 (yellow), and THP-1 M0 (brown) nPMVs.

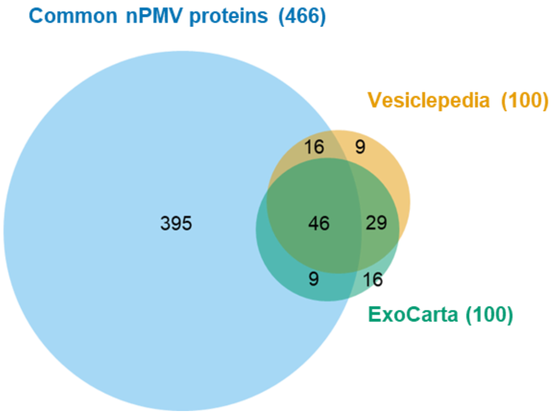

Supplementary Figure 9: Common nPMV proteins in comparison to EV and exosome proteins listed in databases presented as a Venn diagram. The 466 common nPMV proteins (blue) were compared to top 100 proteins of the Vesiclepedia [1,2] (orange) and ExoCarta [3,4] (green) databases. Size of the circle is representative for the total counts. Overlapped circle area is representative for the overlap counts.

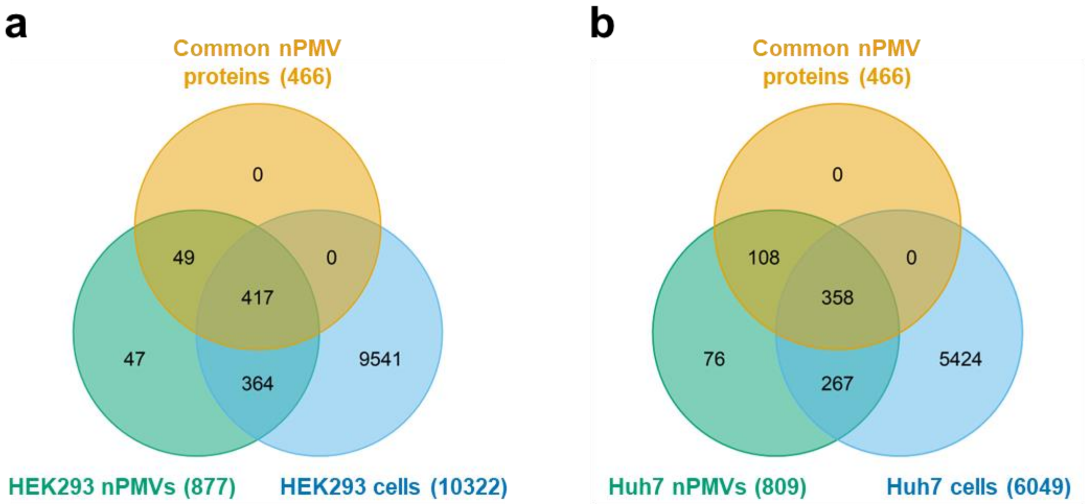

Supplementary Figure 10: Venn diagram between nPMV, common nPMV, and donor cell proteins. nPMV proteins (green) of HEK293 (**a**) or Huh7 (**b**) were compared to the proteins of the corresponding donor cell (blue) and the common 466 nPMV proteins (orange). Values in paratheses indicate the total counts of each group.

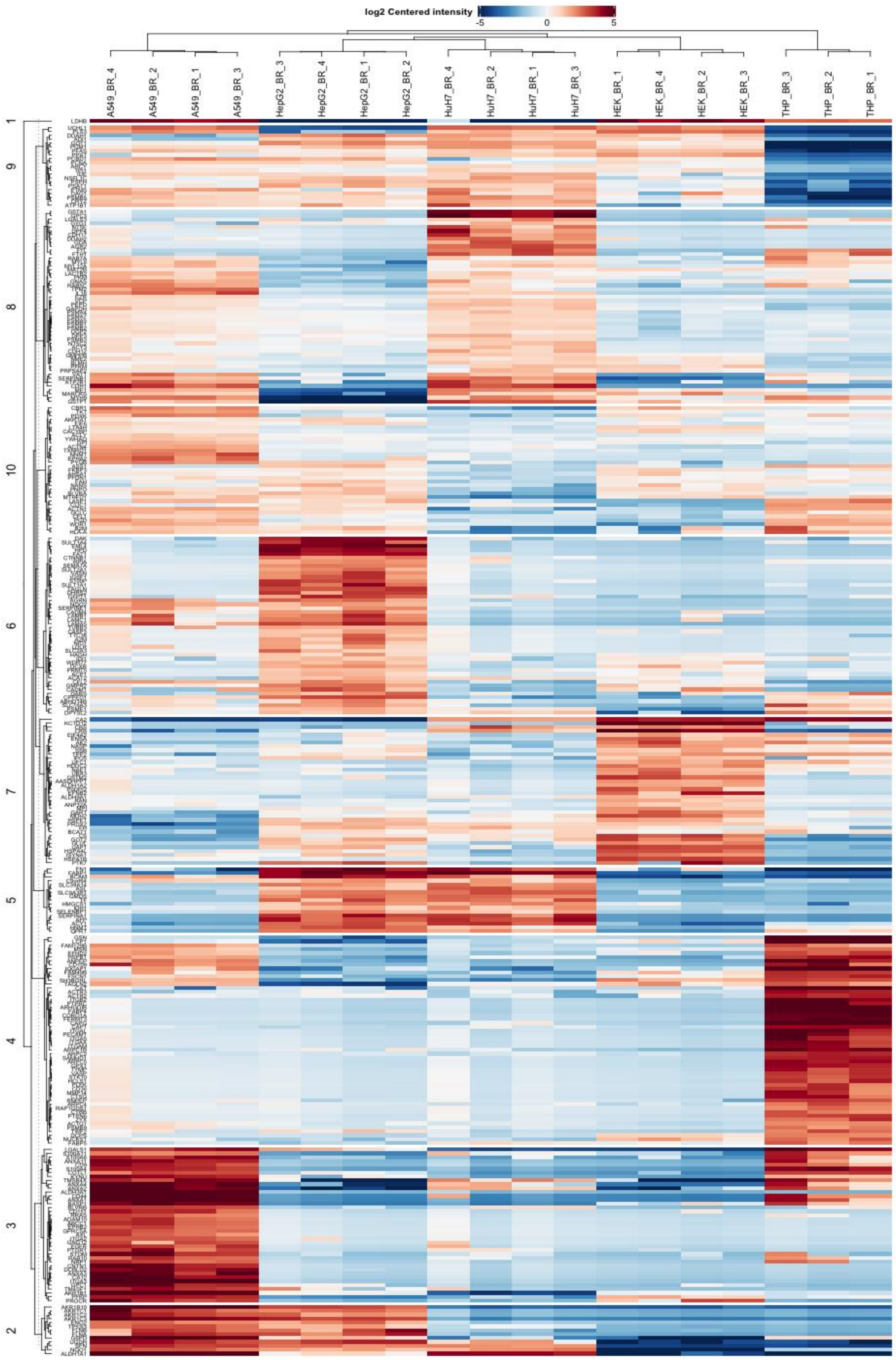

Supplementary Figure 11: Proteomic analysis using a heat map representation of 150 proteins. Analysis of the proteome of all nPMV types (n≥3) allowing for a comparison between the indicated nPMV types. Log_2 values of signal intensities (color map) are shown.

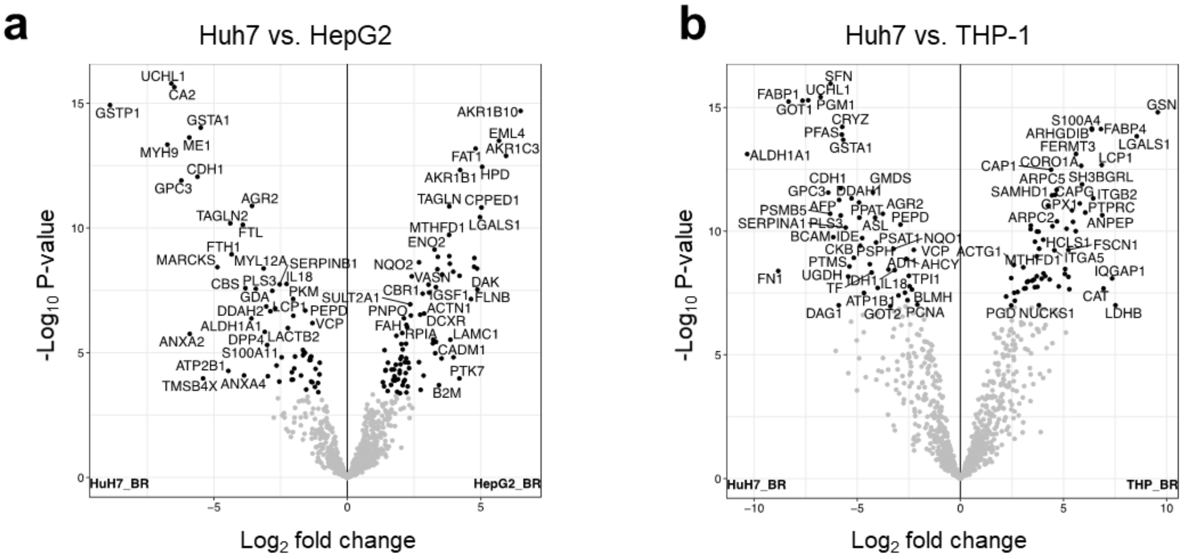

Supplementary Figure 12: Proteomic comparison between different nPMV types. Volcano plot showing differentially expressed proteins between Huh7 and HepG2 (**a**) or THP-1 M0 macrophage (**b**) nPMVs (n≥3). The top hits are marked in bold.

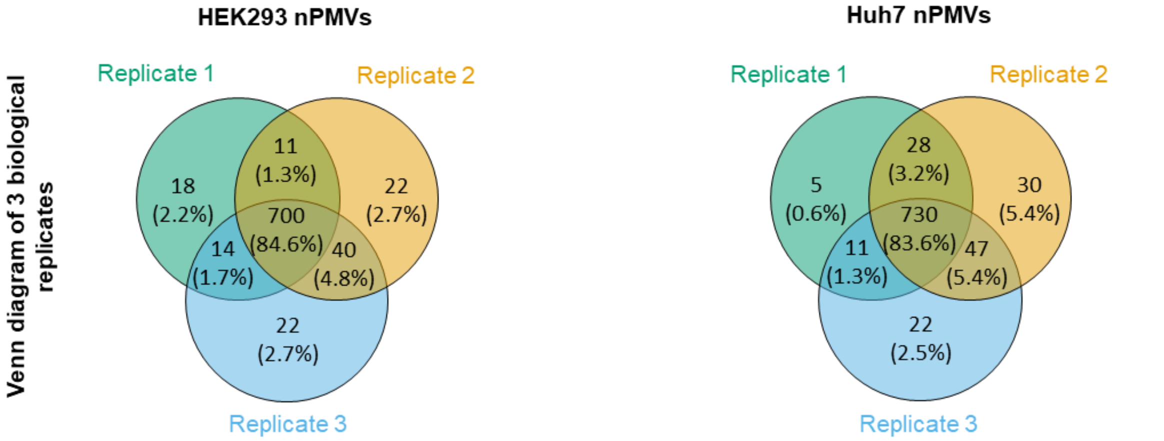

Supplementary Figure 13: Venn diagrams of nPMV proteomes from 3 biological replicates. Venn diagram of replicate 1 (green), 2 (orange), and 3 (blue) of HEK293 (left) or Huh7 (right) nPMVs indicated an overlap of >83% between biological replicates. Values in paratheses indicate the percentage value of the total counts.

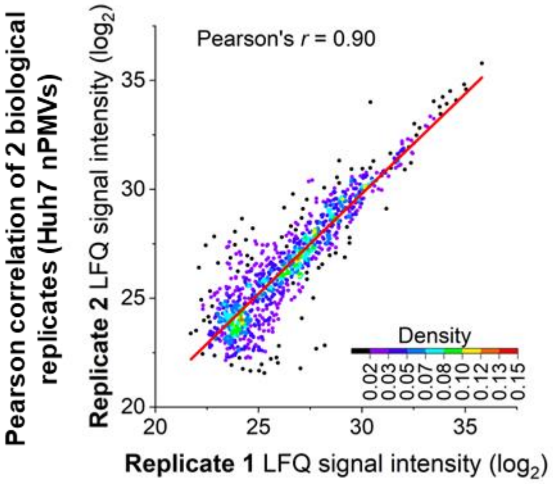

Supplementary Figure 14: Pearson correlation between the biological replicate 1 and 2 of Huh7 nPMVs. The Pearson correlation between the label-free quantification (LFQ) of biological replicate 1 and 2 of Huh7 nPMVs. The whole proteome was used for the analysis. Color map: indicates the dot plot density. Red line: linear fit of the data.

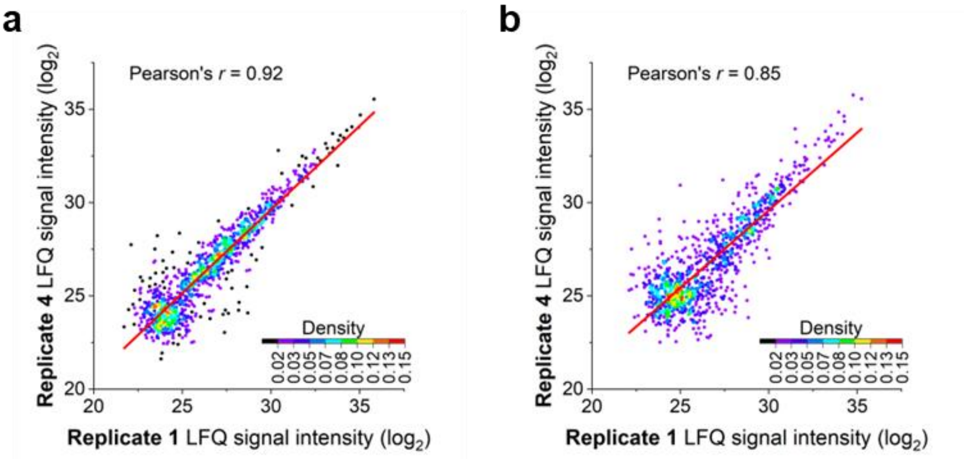

Supplementary Figure 15: Pearson correlation between the biological replicate 1 and 4 of the nPMV proteomes. The Pearson correlation between the label-free quantification (LFQ) of biological replicate 1 and 4 of HEK293 (**a**) and Huh7 nPMVs (**b**). The whole proteome was used for the analysis. Color map: indicates the dot plot density. Red line: linear fit of the data.

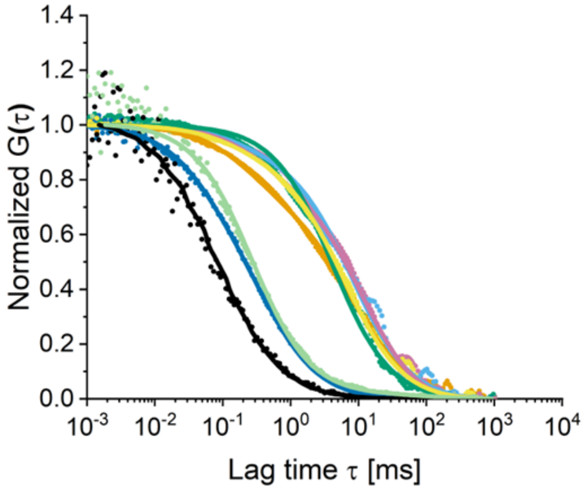

Sample name	Diffusion coefficient D [$\mu m^2/s$]
HEK293 nPMVs	3.7 ± 0.03
HEK293 native EVs	5.8 ± 0.03
Huh7 nPMVs	6.2 ± 0.01
Huh7 native EVs	3.7 ± 0.03
DOPC:PS liposomes	5 ± 0.02
DOPC liposomes	95 ± 0.01
Annexin V Alexa Fluor 488	90 ± 0.01

Supplementary Figure 16: Fluorescence correlation spectroscopy (FCS) analysis of indicated NPs after incubating with annexin V Alexa Fluor 488. HEK293 nPMVs (green), HEK293 native EVs (orange), Huh7 nPMVs (blue), Huh7 native EVs (pink), DOPC:PS liposomes (yellow), and DOPC liposomes (dark blue) were incubated with annexin V Alexa Fluor 488 (light green) and analyzed by FCS. Diffusion coefficient D for each sample is shown in the table on the right. Calibration was performed with Atto 488 carboxylic acid (black).

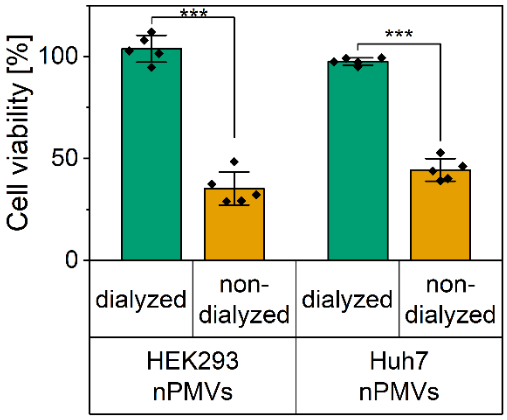

Supplementary Figure 17: Cell viability of Huh7 cells. Significantly lower cell viability of Huh7 cells were observed 24 h after incubation with not dialyzed compared to dialyzed HEK293 and Huh7 nPMVs. Values are means ± SD, squares: data points, n=5 measurements. Levels of significance: *: $p \leq 0.05$, **: $p \leq 0.01$, ***: $p \leq 0.001$.

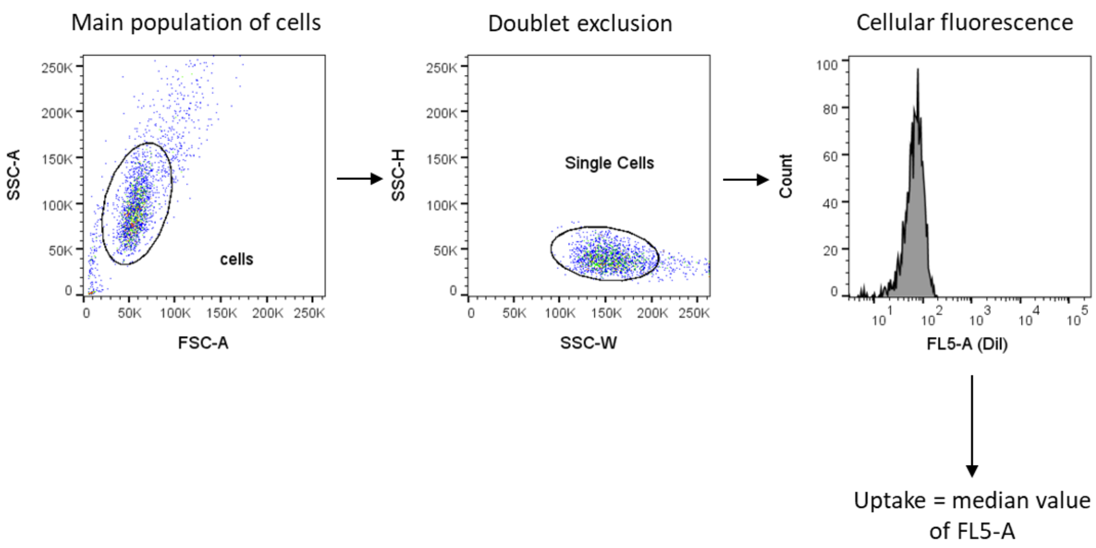

Supplementary Figure 18: Gating strategy for the uptake experiment in Figure 4 b, c. The main population of cells was gated in the FSC-A/SSC-A channels. Cell doublets were further excluded by SSC-W/SSC-H gating. The FL5-A channel served as detection channel for the DiI signal. The uptake of NPs was quantified by the median value of the cellular fluorescence.

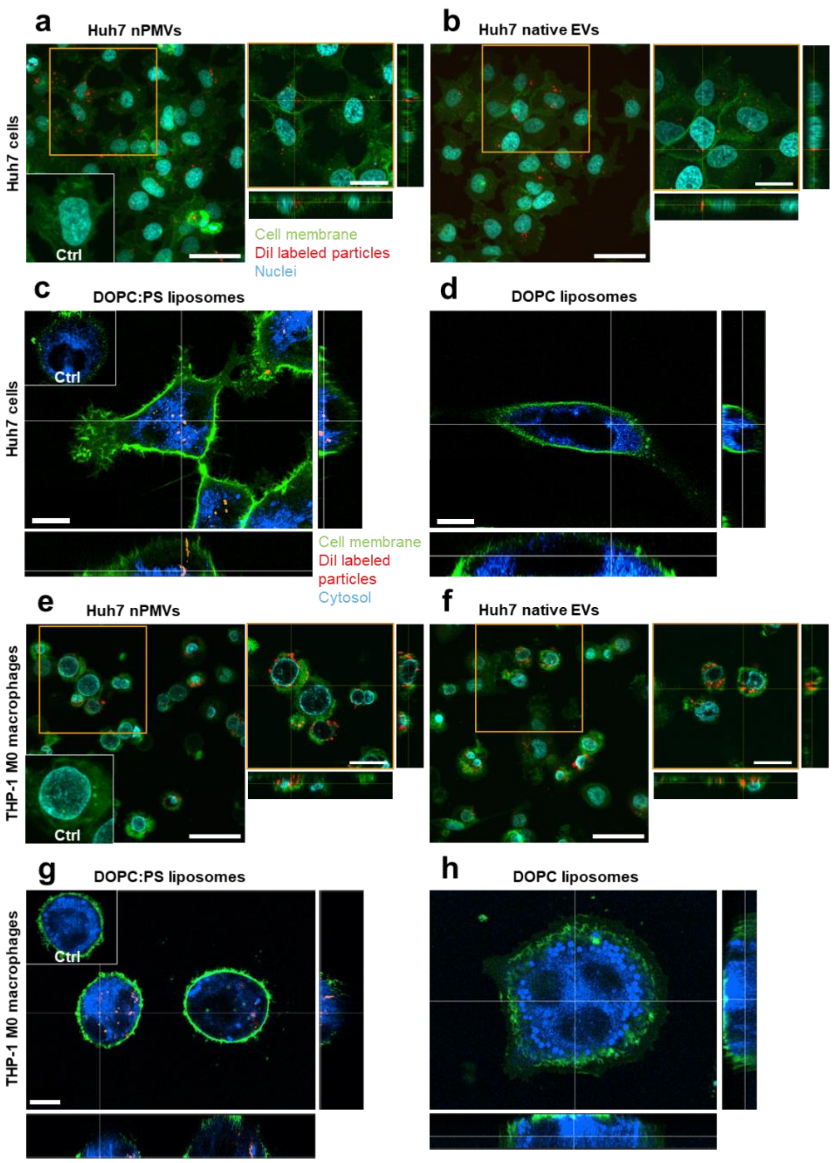

Supplementary Figure 19: CLSM images of indicated NPs incubated with Huh7 cells and THP-1 M0 macrophages. Z-projection of Huh7 cells (top two rows) and THP-1 M0 macrophages (bottom two rows) after 1 h incubation with Huh7 nPMVs (**a, e**), Huh7 native EVs (**b, f**), DOPC:PS (**c, g**), and DOPC liposomes (**d, h**) imaged with CLSM. Experiment setup for Huh7 nPMVs and native EVs: green signal: cell membrane. Red signal: DiI labeled particles. Blue signal: Nuclei. Scale bar: 50 µm. Right panel: Zoomed Z-projection and orthogonal view of the orange indicated region. Scale bar: 25 µm. Experimental setup for DOPC:PS and DOPC liposomes: Blue signal: cytosol. Green signal: cell membrane. Red signal: DiI labeled particles. Scale bar: 10 µm.

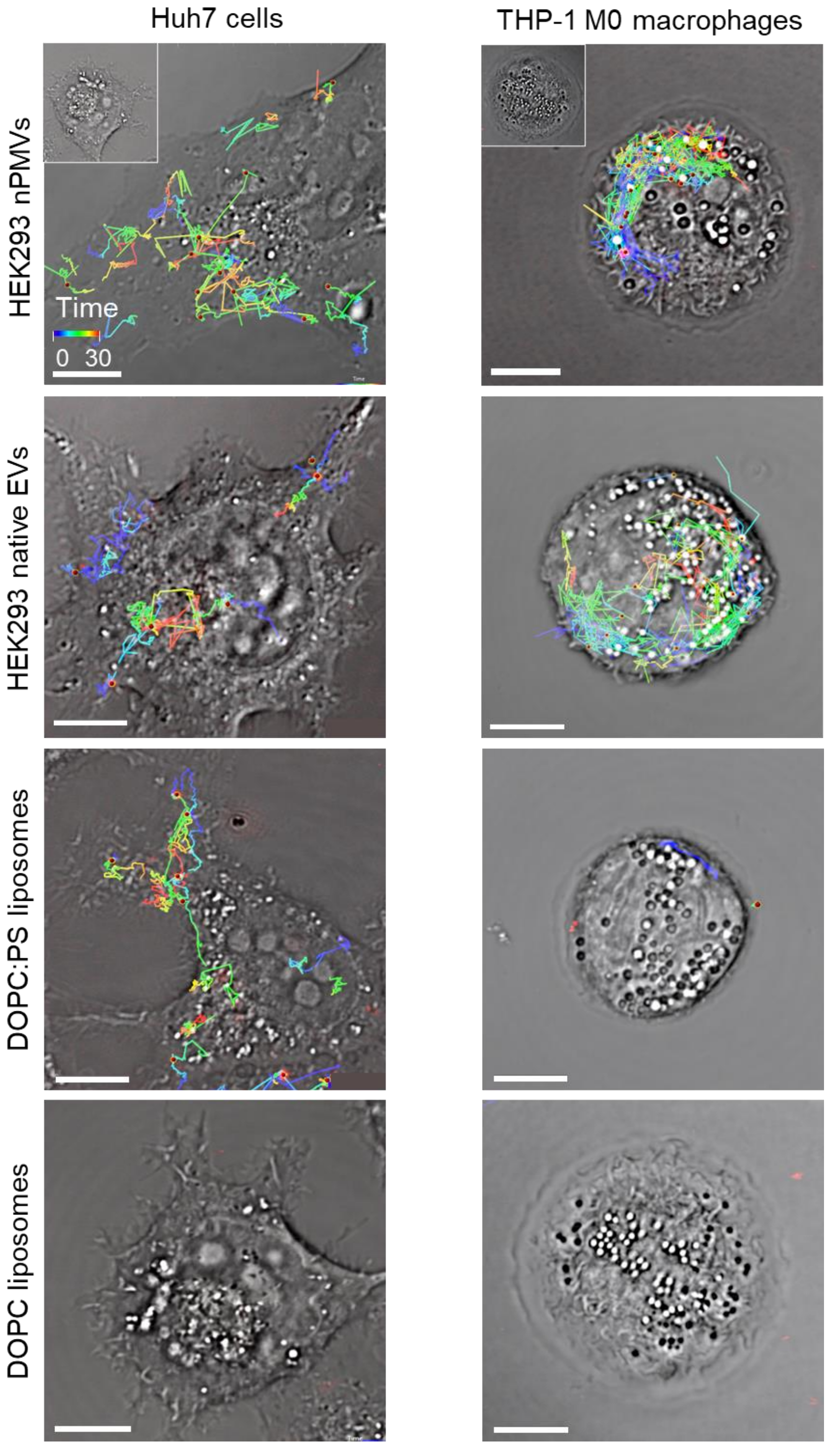

Supplementary Figure 20: Live cell imaging and single particle tracking. Single particle trajectories of indicated DiI labeled NPs with either Huh7 cells (left panel) or THP-1 M0 macrophages (right panel). Images of HEK293 nPMVs and native EVs are represenative for Huh7 nPMVs and native EVs. Insert: untreated cells. Scale bar: 10 µm. Heat mapped single particle trajectories (0-30 minutes).

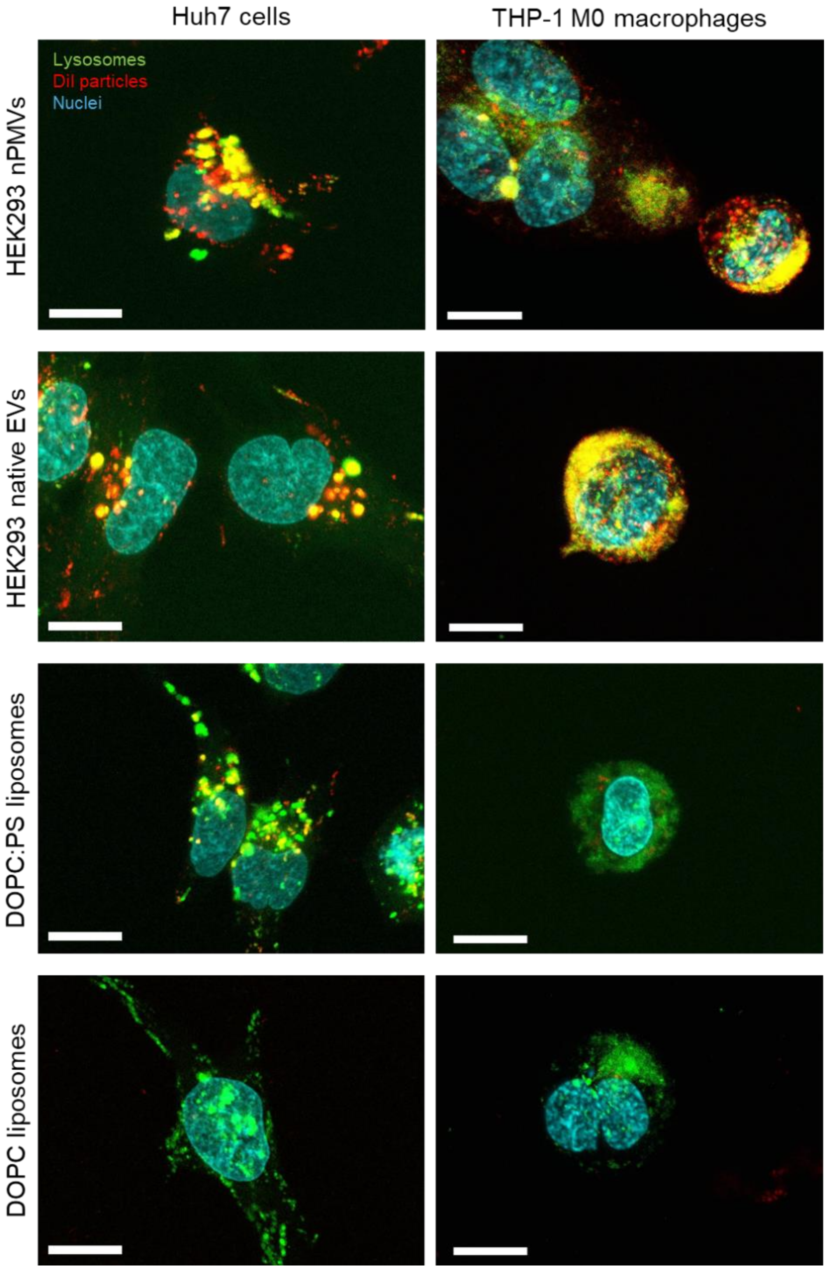

Supplementary Figure 21: Colocalization of NPs with lysosomes in Huh7 cells and THP-1 M0 macrophages. Huh7 (left panel) and THP-1 M0 (right panel) cells were incubated with indicated NPs for 4 h and imaged using CLSM. Images acquired with HEK293 nPMVs and native EVs are representative for Huh7 nPMVs and native EVs. Red signal: DiI labeled NPs. Green signal: Lysotracker Green. Yellow signal: colocalization of red and green signals. Blue signal: Hoechst 33342. Scale bar: 15 µm.

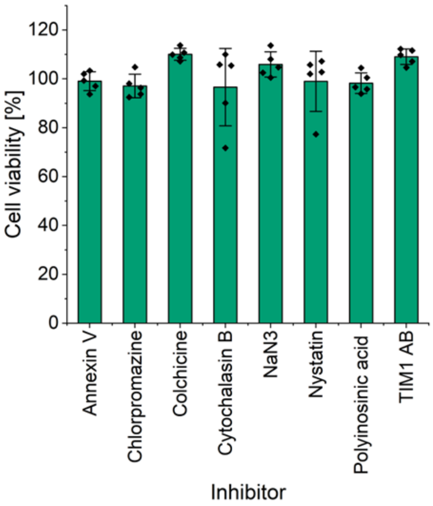

Supplementary Figure 22: Viability of cells treated with indicated inhibitors. Huh7 cells were treated with indicated uptake inhibitors for 24 h before the viability was analyzed with MTS assay. Values are means ± SD, squares: data points, n=5 measurements.

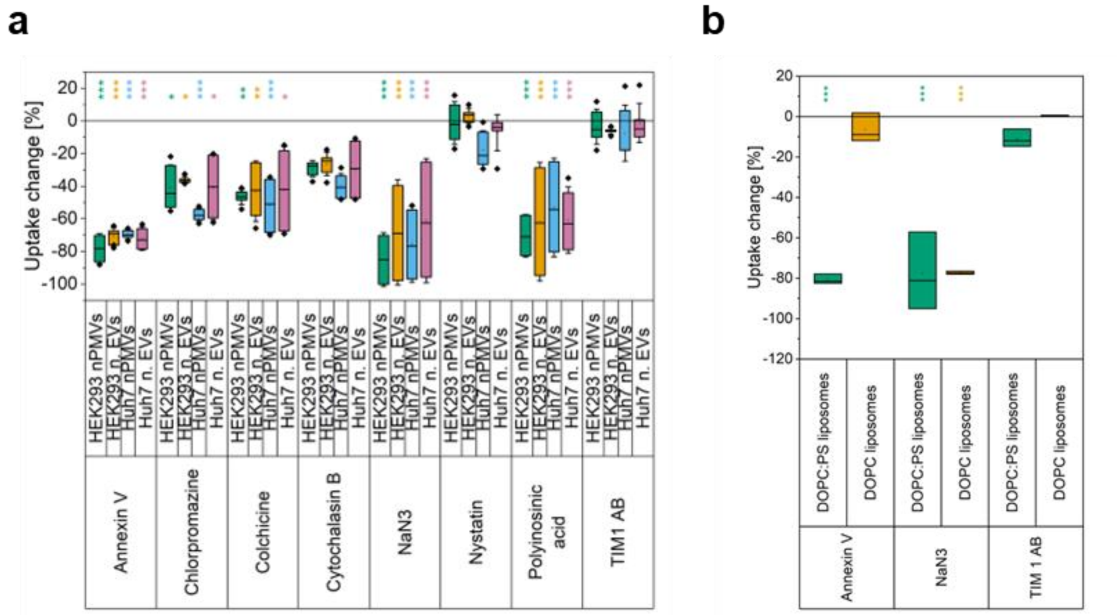

Supplementary Figure 23: Impact of indicated uptake inhibitors on the internalization of NPs in Huh7 cells. a: HEK293 nPMVs (green), HEK293 native EVs (orange), Huh7 nPMVs (blue), and Huh7 native EVs (pink). **b:** DOPC:PS (green) and DOPC (orange). Box plot: line: median, square: mean, box: lower and upper quartile, whisker: 1.5 interquartile range, filled square: outlier, n≥3. Levels of significance: *: p ≤ 0.05, **: p ≤ 0.01, ***: p ≤ 0.001.

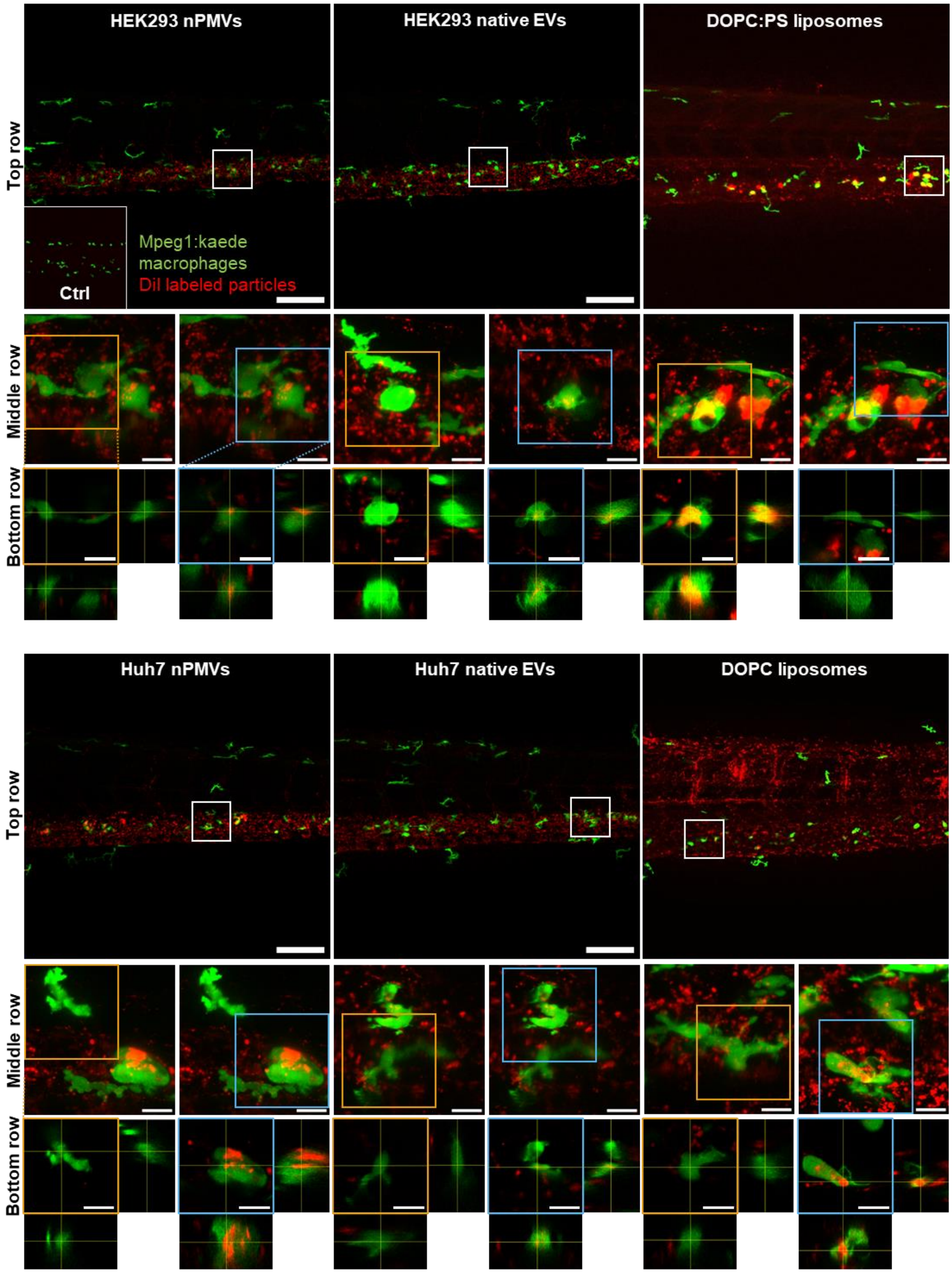

Supplementary Figure 24: *In vivo* biodistribution of HEK293 and Huh7 nPMVs and native EVs, DOPC:PS, and DOPC liposomes in the zebrafish larvae (ZFL). Top rows: Tissue distribution of the indicated NPs in 48 hpf old Tg(mpeg1:Gal4:UAS:Kaede) ZFE imaged with

CLSM 24 hpi. Green signal: mpeg1:Gal4:UAS:Kaede (macrophages). Red signal: DiI NPs. Scale bar: 100 µm. Insert: untreated ZFE. Middle rows: Zoomed in Z-projection of the white indicated region in the images of the top rows. Scale bar: 10 µm. Bottom rows: Z-projection and orthogonal views of an empty (orange indicated region) and filled (blue indicated region) macrophage per NP formulation. Scale bar: 10 µm.

Supplementary Tables

Supplementary Table 1: Calculation of the production yield and rate of the nPMV and native EV preparation protocol. Production rate and yield of the nPMV and native EV preparation protocol were calculated using the number of donor cells, production time, particle concentration (measured by NTA), and total sample volume.

Sample name	# of donor cells [x10^{7}]	Production time [h]	Particle concentration [x10^{11}/mL]	Total sample volume [mL]	Particles per cell [#]	Particles per cell and hour [#/h]
HEK293 nPMVs	2	6	1.1	1.2	6600	1100
HEK293 native EVs	16	48	1.19	0.36	267	5
Huh7 nPMVs	2.5	6	2.08	1.0	8320	1386
Huh7 native EVs	16	48	0.98	0.5	308	6

Supplementary Table 2: nPMV associated proteins according to function as identified by proteomics. Summary of the common nPMV proteins of all five nPMV types used in the present study. Proteins are shown, which are known to be present in EVs [2,4–6].

	Protein	Gene name
**chaperones**	Histone H4	HIST1H4A
	Heat shock protein HSP 90-alpha	HSP90AA1
	Heat shock protein HSP 90-beta	HSP90AB1

	78 kDa glucose-regulated protein	HSPA5
	Heat shock cognate 71 kDa protein	HSPA8
	T-complex protein 1 subunit beta	CCT2
	T-complex protein 1 subunit gamma	CCT3
	T-complex protein 1 subunit delta	CCT4
	T-complex protein 1 subunit zeta	CCT6A
	T-complex protein 1 subunit theta	CCT8
	T-complex protein 1 subunit alpha	TCP1
**14-3-3 proteins (Family of conserved regulatory adaptor molecules)**	14-3-3 protein beta/alpha	YWHAB
	14-3-3 protein epsilon	YWHAE
	14-3-3 protein gamma	YWHAG
	14-3-3 protein theta	YWHAQ
	14-3-3 protein zeta/delta	YWHAZ
**cytoskeletal proteins**	Alpha-actinin-1	ACTN1
	Alpha-actinin-4	ACTN4

	Cofilin-1	CFL1
	Moesin	MSN
	Adenylyl cyclase-associated	
	protein 1	CAP1
	Ezrin	EZR
	Profilin-1	PFN1
	Tubulin alpha-1B chain	TUBA1B
	Tubulin beta-4B chain	TUBB4B
**annexins**	Annexin A2	ANXA2
	Annexin A5	ANXA5
**enzymes**	Fructose-bisphosphate	
	aldolase A	ALDOA
	Alpha-enolase	ENO1
	Glyceraldehyde-3-	
	phosphate dehydrogenase	GAPDH
	Phosphoglycerate kinase 1	PGK1
	Pyruvate kinase PKM	PKM
	ATP-citrate synthase	ACLY
	Adenosylhomocysteinase	AHCY
	Sodium/potassium-	
	transporting ATPase subunit	
	alpha-1	ATP1A1
	Fatty acid synthase	FASN

	Glucose-6-phosphate isomerase	GPI
	L-lactate dehydrogenase A chain	LDHA
	Peptidyl-prolyl cis-trans isomerase A	PPIA
	Triosephosphate isomerase	TPI1
	Transitional endoplasmic reticulum ATPase	VCP
	Elongation factor 2	EEF2
	Eukaryotic initiation factor 4A-I	EIF4A1
**peroxiredoxins**	Peroxiredoxin-1	PRDX1
**transport proteins**	Chloride intracellular channel protein 1	CLIC1
	Importin subunit beta-1	KPNB1
	GTP-binding nuclear protein Ran	RAN
	4F2 cell-surface antigen heavy chain / CD98	SLC3A2
	Transferrin receptor protein 1 / CD71	TFRC
**signaling**	Basigin / CD147	BSG

**modulators**	Elongation factor 1-alpha 1	EEF1A1
	Rab GDP dissociation inhibitor beta	GDI2
**membrane trafficking**	Ras-related protein Rab-7a	RAB7A
	Keratin, type II cytoskeletal 1	KRT1

Supplementary Table 3: DAMPs proteins in nPMVs. Summary of DAMPs proteins found in the common nPMV proteins, which are known to be present in EVs [7].

**Origin**		**DAMP proteins**
**Extracellular matrix**		Fibronectin
**Cytosol**	S100 proteins	Calcyclin-binding protein
		Protein S100-A9
	Heat shock proteins	Heat shock protein HSP 90-beta
		Heat shock cognate 71 kDa protein
		Heat shock protein HSP 90-alpha
		Heat shock 70 kDa protein 4
		Heat shock protein 105 kDa
		78 kDa glucose-regulated protein
		60 kDa heat shock protein, mitochondrial
		Endoplasmin
		10 kDa heat shock protein, mitochondrial
		Activator of 90 kDa heat shock protein ATPase homolog 1
		Putative heat shock protein HSP 90-beta 2
		Heat shock 70 kDa protein 6
		Heat shock 70 kDa protein 6

		78 kDa glucose-regulated protein
	F-actin components	F-actin-capping protein subunit alpha-1<br>F-actin-capping protein subunit beta<br>F-actin-capping protein subunit alpha-2<br>Alpha-actinin-1
		Cyclophilin A /Peptidyl-prolyl cis-trans isomerase A
**Nuclear**	Histones	Histone H2B<br>Non-histone chromosomal protein HMG-17<br>Histone H4<br>Histone-binding protein RBBP4<br>Histone H2A<br>Protein arginine N-methyltransferase 5<br>Protein arginine N-methyltransferase 1<br>Histone H3.2
		High mobility group protein B1
**Endoplasmic reticulum**		Calreticulin
	Peroxiredoxins	Peroxiredoxin-6<br>Peroxiredoxin-1<br>Peroxiredoxin-2

Supplementary Table 4: NP brightness as determined by FCS. The brightness of indicated DiI labelled NPs was measured with FCS and used for the uptake normalization.

SAMPLE NAME	BRIGHTNESS [CPM]	NORMALIZATION FACTOR (BASED ON LOWEST BRIGHTNESS)
HEK293 nPMVs	409831	0.891
HEK293 native EVs	443477	0.823
Huh7 nPMVs	495940	0.736
Huh7 native EVs	435743	0.838
DOPC:PS liposomes	365132	1
DOPC liposomes	534539	0.683

Supplementary References

1. Pathan, M. *et al.* Vesiclepedia 2019: a compendium of RNA, proteins, lipids and metabolites in extracellular vesicles. *Nucleic Acids Res.* **47**, D516–D519 (2019).
2. Vesiclepedia: http://microvesicles.org/.
3. Keerthikumar, S. *et al.* ExoCarta: A Web-Based Compendium of Exosomal Cargo. *J. Mol. Biol.* **428**, 688–692 (2016).
4. ExoCarta: http://www.exocarta.org/.
5. Kugeratski, F. G. *et al.* Quantitative proteomics identifies the core proteome of exosomes with syntenin-1 as the highest abundant protein and a putative universal biomarker. *Nat. Cell Biol.* **23**, 631–641 (2021).
6. Rontogianni, S. *et al.* Proteomic profiling of extracellular vesicles allows for human breast cancer subtyping. *Commun. Biol.* **2**, 1–13 (2019).
7. Roh, J. S. & Sohn, D. H. Damage-Associated Molecular Patterns in Inflammatory Diseases. *Immune Netw.* **18**, (2018).
